# Therapeutic potential of Sheng-Xian-Tang in doxorubicin-induced chronic heart failure by regulation of phenylalanine metabolism disruption

**DOI:** 10.1186/s13020-025-01316-6

**Published:** 2026-01-12

**Authors:** Tao Pang, Chao Wang, Guangyang Jiao, Xiangcheng Fan, Doudou Huang, Zhimin Long, Mengqing Xiao, Lianna Sun, Wansheng Chen, Feng Zhang

**Affiliations:** 1 Department of Pharmacy, Changzheng Hospital, Naval Medical University (Second Military Medical University), Shanghai, 200003 China; 2 The SATCM Key Laboratory for New Resources & Quality Evaluation of Chinese Medicine, Institute of Chinese Materia Medica, Shanghai University of Traditional Chinese Medicine, Shanghai, 201203 China; 3 Tongde Hospital of Zhejiang Province Affiliated to Zhejiang Chinese Medical University, (Tongde Hospital of Zhejiang Province), Hangzhou, 310014 Zhejiang China; 4 Zhejiang Academy of Traditional Chinese Medicine, Hangzhou, 310014 Zhejiang China; 5 School of Pharmacy, Shanghai University of Traditional Chinese Medicine, Shanghai, 201203 China; 6 AB SCEIX, Shanghai, 200050 China; 7 Shanghai Key Laboratory for Pharmaceutical Metabolite Research, Naval Medical University (Second Military Medica University), Shanghai, 200433 China

**Keywords:** Doxorubicin, Cardiotoxicity, Sheng-Xian-Tang, Metabolomics, Microbiome, Phenylalanine hydroxylase

## Abstract

**Background:**

Sheng-Xian-Tang (SXT), a traditional Chinese medicine, ameliorates doxorubicin (DOX)-induced chronic heart failure (CHF), yet its molecular mechanisms remain elusive.

**Objective:**

To elucidate SXT's cardioprotective mechanisms against DOX-induced CHF.

**Methods:**

In vivo, cardioprotection was evaluated via echocardiography, oxidative stress assays, and histopathology. Integrated metabolomic and 16S rRNA sequencing identified metabolic disruptions. Serum pharmacochemistry analysis identified hepatic bioactive compounds targeting phenylalanine hydroxylase (PAH). Molecular docking, CETSA, SPR, and enzyme activity assay validated neomangiferin-PAH interactions.

**Results:**

SXT dose-dependently improved DOX-induced cardiac dysfunction in rats. Metabolomic and microbiome analyses confirmed phenylalanine metabolic disorder in the CHF rats. DOX exposure elevated phenylalanine levels in plasma, urine, and heart, reducing hepatic PAH expression and function while inducing ectopic phenylalanine catabolism in the heart. Phenylalanine administration exacerbated the cardiac abnormalities, whereas SXT effectively prevented attenuated DOX-induced cardiac toxicity. CETSA and SPR revealed a strong binding of neomangiferin to PAH, stabilizing its interaction with cofactor BH_4_ and preventing DOX-induced PAH inhibition.

**Conclusions:**

SXT mitigated DOX-induced CHF through hepatic PAH modulation. Neomangiferin could enhance PAH stability via competitive binding. Targeting PAH-phenylalanine metabolism emerged as a novel therapeutic strategy for DOX-induced cardiac dysfunction.

**Graphical Abstract:**

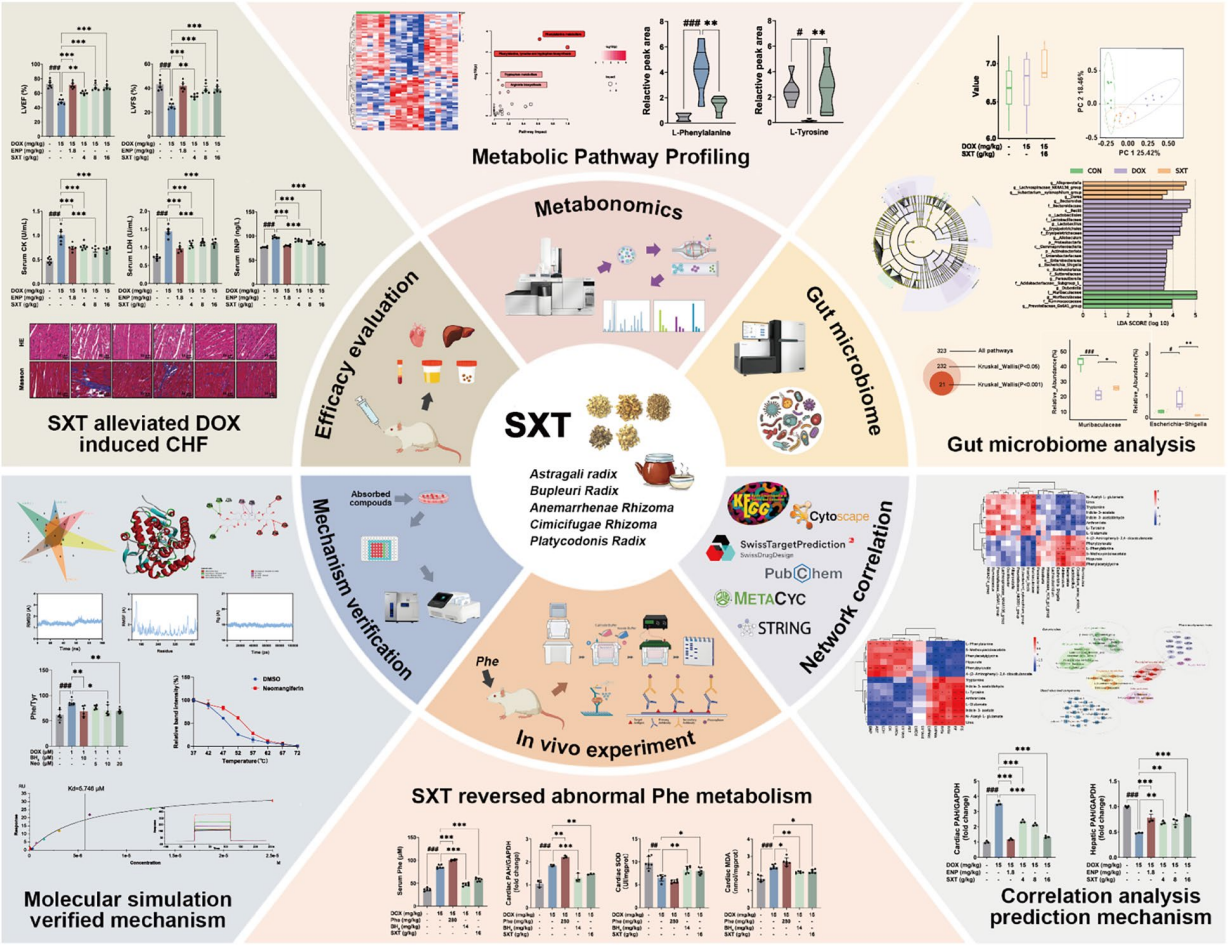

**Supplementary Information:**

The online version contains supplementary material available at 10.1186/s13020-025-01316-6.

## Introduction

Doxorubicin (DOX) is an effective chemotherapeutic agent against a variety of cancers. However, long-term DOX use can cause cardiotoxicity such as dose-dependent chronic heart failure (CHF) and subsequent irreversible heart damage, which restricts its clinical application [26, 28, 38]. Over the past half century, several cellular and molecular mechanisms have been proposed to explain DOX-induced cardiotoxicity, including mitochondrial dysfunction, DNA damage response, inflammatory cytokines, oxidative stress, as well as various forms of regulated cell death, yet effective treatments remain elusive [11, 18, 39]. These multifarious pathophysiological alterations collectively orchestrate the deterioration of cardiac architecture and function, making the development of specific intervention for DOX-induced cardiotoxicity as one of the greatest unmet needs in clinical practice.

Natural products and Chinese medicine may well be a solution for ameliorating DOX-induced cardiotoxicity, because it has great advantages in multiple effects on the pathophysiological alterations involved [19, 31, 41, 47]. Sheng-Xian-Tang (SXT) is one of the representative TCM formulas for CHF treatment, developed by the famous TCM physician Zhang Xi-chun. It is composed of *Astragali Radix*, *Bupleuri Radix*, *Cimicifugae Rhizoma*, *Anemarrhenae Rhizoma* and *Platycodonis Radix* with a dose proportion of 6:1.5:1:3:1.5. Modern pharmacological research has shown that the individual herbs in SXT exhibit efficacy in anti-inflammation, anti-oxidation, anti-fibrosis, promoting angiogenesis, enhancing immunity and other functions [21, 34, 43]. Our previous study showed that SXT demonstrated a therapeutic effect against DOX-induced cardiomyocyte injury in vitro and in vivo, but the underlying mechanism remained unknown [14, 22].

Metabolomics can provide an unbiased and comprehensive qualitative and quantitative overview of endogenous metabolites and their dynamic changes in biological fluids, demonstrating the high potential role of these small molecule metabolites in biological processes [35]. It is widely applied in discovering disease-associated markers and monitoring drug therapy responses [2]. Furthermore, with the development of microbiology, many studies have observed alterations in the compositions of gut microbiota, using 16S ribosomal RNA (rRNA) gene sequencing in patients with chronic heart failure [46], with emerging links between metabolomics and gut microbiota presenting a promising target for studying the disease [9].

This study systematically investigated the therapeutic effect of SXT on DOX-induced CHF. A urinary metabolomics and 16S rRNA gene sequencing were conducted to explore the molecular mechanism. The target validation techniques drug affinity responsive target sability (DARTS) and cellular thermal shift assay (CETSA) were applied to elucidate the molecular mechanisms underlying SXT's cardioprotective effects, for the absorbed compounds by the liver from SXT.

## Materials and methods

### Reagents

*Astragali Radix*, *Anemarrhenae Rhizoma*, *Cimicifugae Rhizoma*, *Bupleuri Radix*, and *Platycodonis Radix* were purchased from Wujiang Shanghai Cai Tong De Tang Chinese Herbal Pieces Co., Ltd. (Jiangsu, China), which were prepared into SXT according to a previously established method (Table S1, Figure S1) [17]. DOX injection (batch no. 2204E3) was purchased from Shenzhen Main Luck Pharmaceuticals Inc. (Shenzhen, China). Enalapril (batch no. 21010512) was obtained from Yangzijiang Pharmaceutical Group Jiangsu Pharmaceutical Co., Ltd. (Jiangsu, China).

Neomangiferin (HY-N0723), calycosin-7-O-β-D-glucoside (HY-N0520), azelaic acid (HY-B0704), isoferulic acid (HY-N0761), timosaponin B-II (HY-N0812), ononin (HY-N0270), daidzein (HY-N0019R), calycosin (HY-N0519), astragaloside IV (HY-N0431), anemarrhenasaponin I (HY-N4213), formononetin (HY-N0183), timosaponin A-III (HY-N0810), timosaponin A-II (HY-N7614) and doxorubicin (HY-15142A) were purchased from MedChemExpress (MCE, United States). Cimigenol xyloside (FY1621-B) was purchased from Nantong FeiYu biological technology Co., LTD., and tetrahydrobiopterin (BH_4_, E065948) was purchased from Shanghai Yifei Biotechnology Co., Ltd. The purity of each compound was higher than 98% as determined by HPLC analysis.

Methanol, acetonitrile, and formic acid (MS grade) were purchased from Merck (Merck Co., Inc., Germany). Ammonium acetate (LC grade) was purchased from Fisher Scientific (Fair Lawn, New Jersey, USA). All other materials and solvents were of analytical grade.

### Doxorubicin (DOX)-induced chronic heart failure (CHF) rat model and sample collection

SD male rats (200–250 g) were obtained from Vital River Laboratory Animal Technology Co., Ltd. (Beijing, China). All animals were raised under specific pathogen-free conditions at 20 ± 2 °C, 55–65% humidity and a 12 h light/dark cycle. The experiment was performed in accordance with the NIH Guide for the Care and Use of Laboratory Animals and approved by the Institutional Animal Care and Use Committee of Shanghai Traditional Chinese University (No. PZSHUTCM200814024, No. PZSHUTCM2304250001). All animals were allowed free access to food and water throughout the experiment.

Experiment I, 48 rats were randomly divided into six groups: (1) the CON group; (2) the DOX group; (3) the DOX + enalapril (ENP, positive control, 1.8 mg/kg) group; (4) the DOX + SXT at low dose group (4 g/kg); (5) the DOX + SXT at middle dose group (8 g/kg); and (5) the DOX + SXT at high dose group (16 g/kg). Except those rats in the CON group, the remaining rats received DOX at a 2.5 mg/kg per injection to a cumulative dose of 15 mg/kg. The dose of SXT for the low-dose group was set at 4 g/kg, based on the minimum human clinical daily dose (48.5 g, Table S2) converted to an equivalent rat dose using body surface area normalization (48.5 g/70 kg × 6.3 ≈ 4.4 g/kg, rounded to 4 g/kg). To evaluate a clear dose–response relationship, the medium and high doses were established at 8 g/kg and 16 g/kg, respectively, corresponding to 2 fold and 4 fold the low dose. This gradient dosing strategy is consistent with the Technical Guidelines for Pharmacodynamic Studies of Traditional Chinese Medicine and the Technical Guidelines for General Pharmacology Studies of Traditional Chinese Medicine and Natural Drugs, and is further supported by our previous study on the effective dose range of SXT in a rat model of doxorubicin-induced chronic heart failure [14, 25, 44]. Heart function was monitored by echocardiography analysis at day 39, and the CHF model was considered to be successfully established when the left ventricular ejection fraction (LVEF%) values were reduced to 50%. Urinary samples were collected at five time points (0, 10, 20, 30, and 39 d), while fecal samples were collected before the rats were euthanized. Subsequently, the rats were anesthetized, and blood samples and heart and liver tissues were collected. All bio-samples were stored at − 80 °C until analysis.

Experiment II was conducted to specifically confirm the exacerbated cardiac abnormality of phenylalanine in the DOX-induced CHF rats. 40 rats were randomly divided into five groups: (1) the CON group; (2) the DOX group; (3) the DOX + phenylalanine group (280 mg/kg); (4) the DOX + BH_4_ group (14 mg/kg); (5) the DOX + SXT (16 g/kg). Heart function was detected as in the experiment I.

### Cardiac functional tests

Echocardiographic (ECG) measurements were performed using M-mode tracings and continuous Doppler with a Vevo 2100 system (Visual Sonics, Toronto, ON, Canada). Rats were anesthetized with isoflurane prior to imaging. Cardiac function indices including left ventricular ejection fraction (LVEF%) and left ventricular fractional shortening (LVFS%) were measured and recorded.

### Histopathological evaluation and apoptosis detection

Cardiac tissues were fixed in 4% paraformaldehyde, embedded in paraffin, and sectioned at 4–5 μm thickness. Hematoxylin and eosin (H&E) staining was performed to assess histopathological changes, while Masson’s trichrome staining was used to evaluate cardiac fibrosis. Apoptosis in cardiac tissues was detected using a TUNEL assay kit (C1088, Beyotime, Shanghai, China). TUNEL-positive apoptotic cells exhibited green fluorescence, while nuclei were counterstained blue with DAPI (4′,6-diamidino-2-phenylindole; Abcam).

### Immunofluorescence staining

Immunofluorescence staining was performed on tissue sections to detect phenylalanine hydroxylase (PAH), prostaglandin-endoperoxide synthase 2 (PTGS2), and reactive oxygen species (ROS). Primary antibodies against PAH and PTGS2 were used, while ROS was detected using the DCFH-DA probe [16]. Nuclei were counterstained with DAPI. Images were captured using a fluorescence microscope.

### Transmission electron microscopy

Freshly excised heart tissues were immediately fixed in 2.5% glutaraldehyde at 4 °C overnight. After washing with 0.1 M cacodylate trihydrate buffer, samples were post-fixed with 1% osmium tetroxide for 1 h. Tissues were then dehydrated through a graded ethanol series, incubated in acetone, and embedded in epoxy resin. Ultrathin sections were mounted on copper grids and imaged using a Hitachi HT7800 transmission electron microscope (Tokyo, Japan).

### ELISA assay

Serum biochemical parameters including brain natriuretic peptide (BNP), creatine kinase (CK), lactate dehydrogenase (LDH), alanine aminotransferase (ALT), and aspartate aminotransferase (AST) were measured using commercial ELISA kits (Nanjing Jiancheng Bioengineering Institute, Nanjing, China) according to the manufacturer’s instructions. Superoxide dismutase (SOD), and malondialdehyde (MDA)  levels in liver and heart tissues were measured using assay kits from Aifang Biotechnology (Changsha, China). Phenylalanine and tyrosine concentrations in the cell were determined using an ELISA kit from Abmart (Shanghai, China).

### Metabolomics analysis

Urine samples (200 μL) were mixed with 200 μL of protein precipitant (methanol: acetonitrile, 1:1) and vortexed for 30 s at 4 °C. Samples were sonicated for 10 min in an ice bath, then incubated at – 20 °C for 1 h. After centrifugation at 13,000 rpm for 15 min at 4 °C, 200 μL of supernatant was transferred to sample vials for analysis. A pooled quality control (QC) sample was prepared by combining 10 μL of supernatant from each sample. Sample analysis was performed using a Waters H-Class UPLC system coupled to a Triple TOF 6600 mass spectrometer (AB SCIEX, USA). Chromatographic separation was achieved on an ACQUITY UPLC HSS T3 column (2.1 × 100 mm, 1.7 μm; Waters) using 0.1% formic acid in water (mobile phase A) and acetonitrile (mobile phase B). The gradient elution program was: 0–1.5 min, 1% B; 1.5–13 min, 1–99% B; 13–16.5 min, 99% B; 16.5–20 min, 1% B. The flow rate was 0.3 mL/min and the injection volume was 2 μL. Mass spectrometry was performed in both positive and negative ionization modes. Key parameters included: ion spray voltage, 5500/− 4500 V; m/z range, 60–1000; gas 1 and 2, 60 psi; curtain gas, 35 psi; accumulation time, 1.2 s; source temperature, 550 °C; declustering potential, 60 V. Information-dependent acquisition (IDA) was used with the following settings: m/z range, 50–1000; accumulation time, 0.05 s; collision energy, 40 ± 20 V. Data processing was performed using XC-MS plus software for peak alignment, extraction, and normalization. Metabolite identification was carried out using the MetDNA database. Multivariate statistical analysis, including principal component analysis (PCA) and orthogonal partial least squares discriminant analysis (OPLS-DA), was conducted using MetaboAnalyst 5.0. Differential metabolites were identified based on variable importance in projection (VIP) scores > 1.0 and P < 0.05. Fold change (FC) values were used to determine up- or down-regulation. Metabolic pathway analysis was performed using MetaboAnalyst 5.0, with significance set at P < 0.05.

### 16S rRNA sequencing and analysis

16S rRNA sequencing and bioinformatics analysis were performed by OEbiotech Co. Ltd. (Shanghai, China). Total genomic DNA was extracted from cecal contents. The V3–V4 hypervariable region of the 16S rRNA gene was amplified using primers 343F and 798R. Clean reads were processed to remove primer sequences and clustered into operational taxonomic units (OTUs) at 97% similarity using Vsearch software. OTUs were annotated by comparison against the UNITE database for fungal ITS sequences [20]. Alpha diversity was assessed using the Simpson index. Beta diversity was evaluated using Bray–Curtis distances and visualized by principal coordinates analysis (PCoA) [37]. Differential abundance analysis of gut microbiota associated with DOX-induced CHF was performed using linear discriminant analysis effect size (LEfSe) and random forest algorithms.

### Cell culture and treatment

Mouse hepatocellular cell lines (AML12), purchased from the Shanghai Cell Bank of Chinese Academy of Sciences (Shanghai, China), were cultured in Dulbecco’s Modified Eagle Medium (DMEM) supplemented with 10% fetal bovine serum (FBS) and 2 mM L-glutamine and 1% pyruvate and maintained at 37 °C in a 95% O_2_, 5% CO_2_ incubator. Cells were seeded at a density of 5 × 10^5^ cells/well in 6-well plates. DOX (1 µM) was selected for the rest of the experiments that caused 50% cell death (IC50). Cells’ survival rate was detected by Cell Counting Kit-8 (CCK8) Reagents (Thermo Scientific, Singapore).

### Western blotting

Protein was extracted from liver or heart tissue samples (30 mg) using lysis buffer. Total protein concentration was determined using a BCA protein assay kit (Thermo Fisher Scientific, USA). Equal amounts of protein from each sample were separated on 10–12% SDS-PAGE gels and transferred to PVDF membranes. Membranes were blocked with quick blocking buffer for 30 min, then incubated with anti-PAH primary antibody (1:1000 dilution) overnight at 4 °C. After washing, membranes were incubated with a secondary antibody for 1 h at 37 °C. Protein bands were visualized using an Odyssey infrared imaging system (JS-1070, Shanghai Peiqing Science & Technology Co., Ltd, China). Band intensities were quantified using ImageJ software.

### Determination of phenylalanine and tyrosine levels in the rat serum, liver and heart tissues

Phenylalanine and tyrosine in the different rat bio-samples were analyzed by LC/MS using an Agilent 1290 Ultra high-performance liquid chromatography (UHPLC) and an Agilent 6460 triple-quadrupole tandem mass spectrometer (QQQ-MS) (Agilent Inc, USA). Data were acquired by using an Agilent Zorbax SB-C18 column (3.0 × 150 mm, 5 μm) as described previously [42].

### Surface Plasmon Resonance (SPR) analysis

SPR assays were conducted using a Biacore T200 system (GE Healthcare Life Sciences, USA). Phenylalanine hydroxylase (PAH) protein was immobilized on CM5 sensor chips using an amine-coupling kit (BR100050, GE Healthcare) following standard procedures [15]. Various concentrations of small molecules, including active compounds from SXT, doxorubicin, or tetrahydrobiopterin, were prepared in running buffer containing 5% DMSO. Samples were injected over reference and PAH-immobilized flow cells at a flow rate of 30 μL/min, with 120 s contact time and 120 s dissociation time. Kinetic parameters were calculated using Biacore T200 evaluation software.

### Cellular Thermal Shift Assay (CETSA)

AML12 cells (5 × 10^5^ cells/well) in 6-well plates were divided into two groups, with 8 wells in each group. (1) DMSO group, where cells were cultured in DMEM/F12 with 0.4% DMSO; (2) neomangiferin group, where cells were cultured in DMEM/F12 with neomangiferin (10 μM). After 24 h, 8 samples of cells from each of the two groups were incubated at specific temperatures (37, 42, 47, 52, 57, 62, 67, and 72 °C) for 3 min and each sample was performed for western blotting analysis.

### Statistical analysis

Data were presented as mean ± standard deviation (SD). Statistical analyses were performed using GraphPad Prism 8.0 (GraphPad Software, Inc., USA). Student’s t-test was used for comparisons between two groups, while one-way analysis of variance (ANOVA) was employed for multiple group comparisons. Kruskal–Wallis tests were used to evaluate differentially abundant gut microbes and serum metabolites. Statistical significance was set at *p* < 0.05 (^#^) or (*), *p* < 0.01 (^##^) or (**), or *p* < 0.001(^###^) or (***). Volcano plots, heatmaps, and box plots were generated using GraphPad Prism.

## Results

### SXT inhibited the DOX-induced cardiac dysfunction in rats

**Fig. 1:**
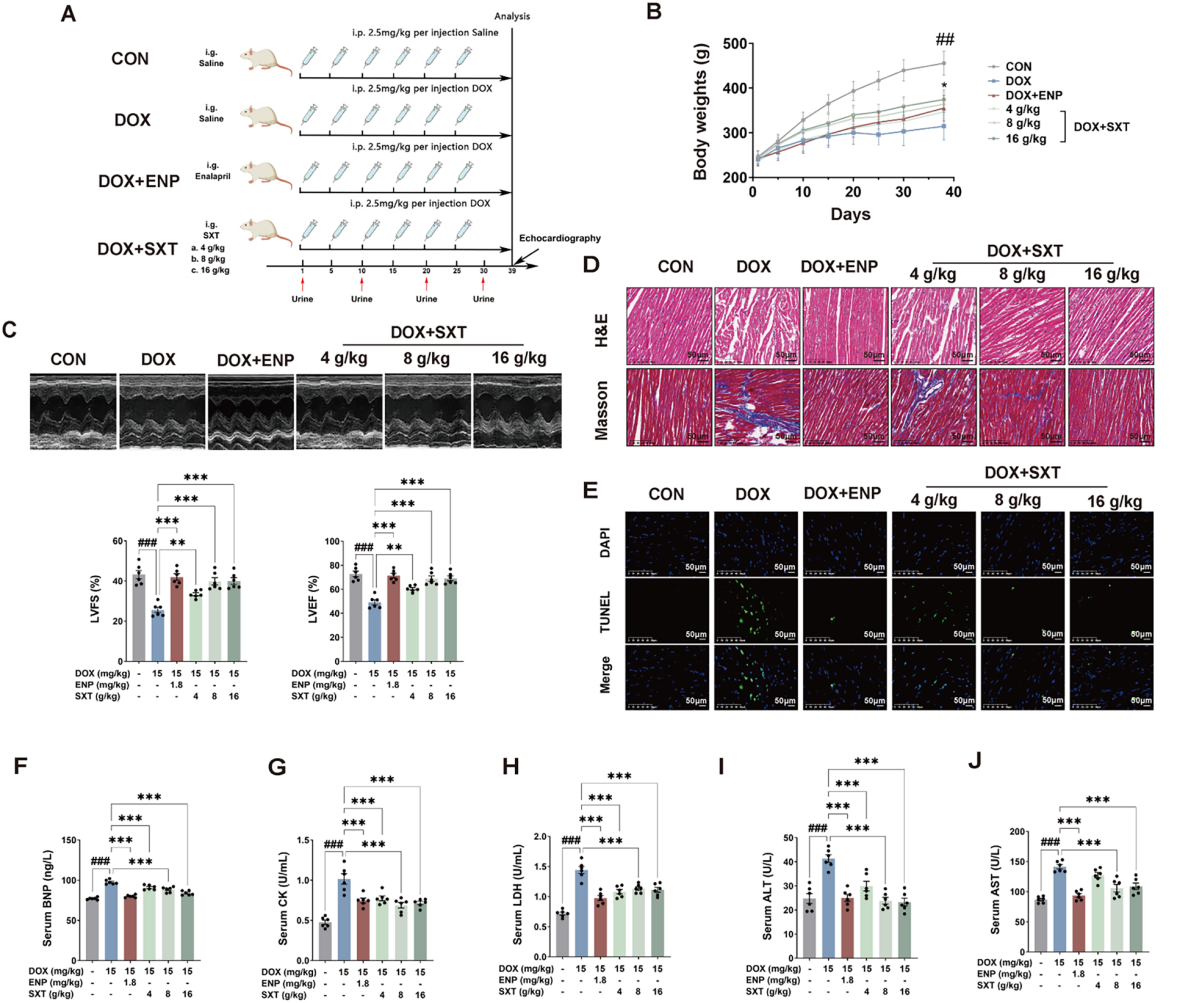
SXT ameliorated DOX‑induced deterioration of cardiac functions in rats. **A** Schematic protocol of the experiment I; **B** rat body weight at different time points; **C** representative sequential measurements of echocardiograms, LVEF % and LVFS %; **D** representative sections of hearts stained with H&E and masson's trichrome for fibrosis detection (blue), scale bars 50 µm; **E** TUNEL stain, scale bars 50 µm; serum levels of (**F**) BNP; **G** CK; **H** LDH; **I** ALT; and (**J**) AST. ^#^*p* < 0.05, ^##^* p* < 0.01 and ^###^*p* < 0.001 vs. the CON group; *p < 0.05, **p < 0.01 and ***p < 0.001 vs. the DOX group

The study design was shown in Fig. 1A. Rat body weight in the control (CON) group showed a steady upward trend, while rats in the other groups had significantly lower body weights compared to the CON group. DOX administration caused a significant reduction in body weight in CHF rats compared with the CON group. Treatment with SXT or ENP partially restored body weight in CHF rats (Fig. 1B). Representative ECG from each group were shown in Fig. 1C. Among the parameters for cardiac function, left ventricular ejection fraction (LVEF) and left ventricular fractional shortening (LVFS) decreased significantly to lower than 50% and 30%, respectively, in the DOX-treated group. As for dose–response data, SXT at doses of 8 and 16 g/kg increased LVEF from 49.17% to 68.89% and 69.15%, and LVFS from 25.62% to 39.85% and 40.02%, respectively, demonstrating its ability to protect rats against DOX-induced CHF (Fig. 1C). The LVEF and LVFS in the ENP group were significantly higher than those in the DOX group (*p* < 0.001). There were no significant differences in these two indices among the ENP, SXT-M (8 g/kg) and SXT-H (16 g/kg) groups (*p* > 0.05).

Histopathological analysis was performed to investigate the therapeutic effects of SXT on cardiac tissue in CHF rats. Hematoxylin and eosin (H&E) and Masson’s trichrome staining indicated that cardiac tissue in the DOX group exhibited inflammatory infiltration and increased collagen deposition, which were significantly attenuated in the SXT-treated groups compared to the DOX group (Fig. 1D). In the CON group, microscopic examination of TUNEL-stained sections revealed sparse single positive nuclei scattered across the ventricular wall. DOX induced nuclear condensation and cytoplasmic shrinkage, consistent with apoptotic morphology. A reduction in TUNEL-positive cardiomyocytes was observed in the ENP and SXT-treated groups (Fig. 1E). In response to 4, 8, and 16 g/kg SXT, a dose-dependent improvement in cardiomyocyte injury was observed, as evidenced by decreased percentages of apoptotic cardiomyocytes.

Additionally, SXT treatment decreased the serum levels of BNP, CK, and LDH in DOX-CHF rats (Figs. 1F–H). Notably, the increased ALT and AST levels in the DOX group were markedly reduced in both SXT- and ENP-treated groups (Figs. 1I and J), indicating that both SXT and ENP could alleviate DOX-induced liver dysfunction.

### SXT alleviated metabolomic disturbance in DOX-induced CHF rats

Metabolomic analysis of urine samples from rats at various time points during the experiment was performed to explore the potential mechanism by which SXT alleviates DOX-induced cardiotoxicity. Figure S2A showed representative total ion chromatograms from UHPLC-Q-TOF/MS analysis of quality control (QC) samples in both positive and negative ionization modes. Principal component analysis (PCA) score plots demonstrate high clustering of QC samples, indicating good reproducibility and data quality (Figure S2B).

**Fig. 2:**
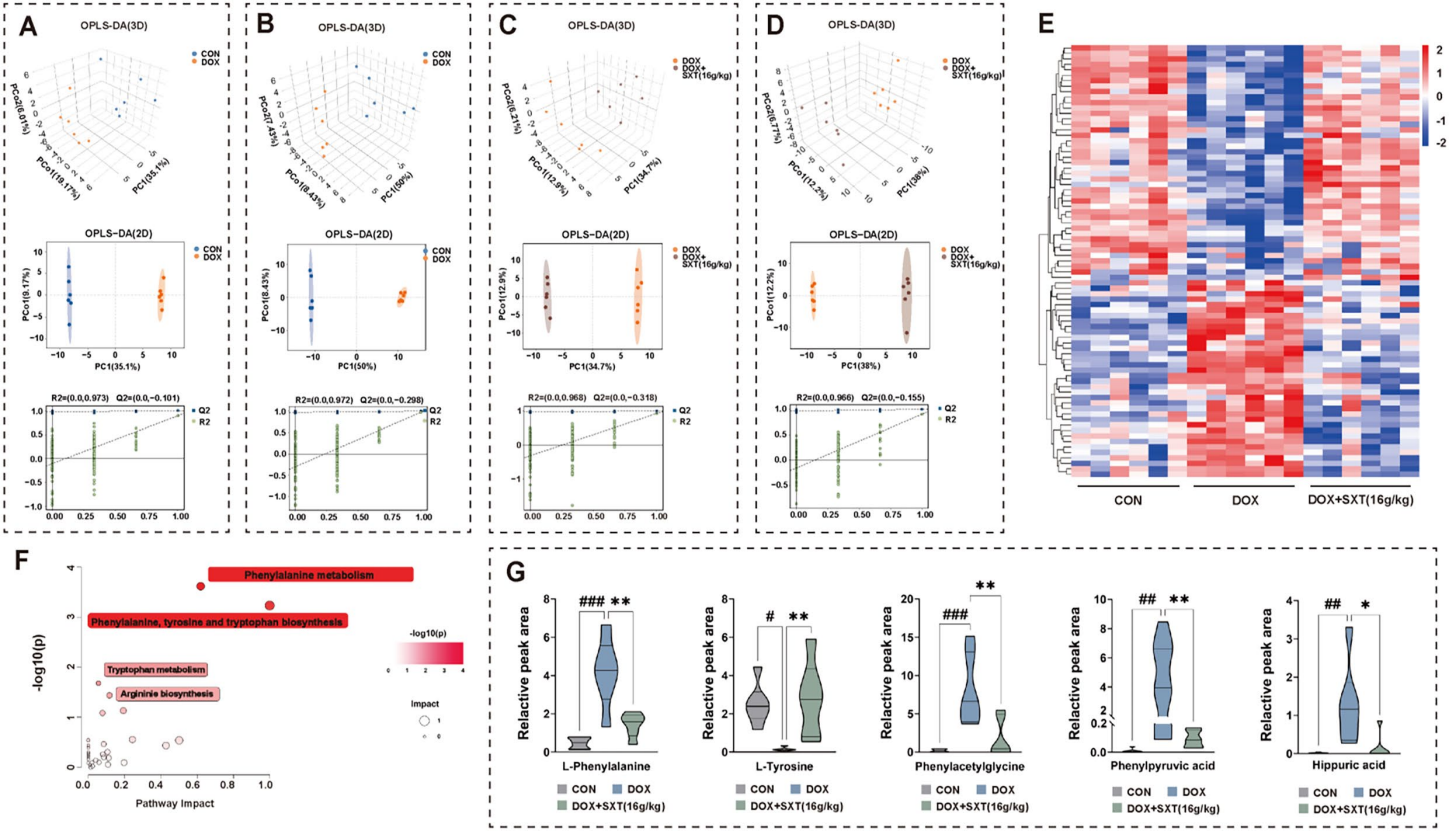
SXT alleviated metabolomic disturbance in DOX-induced CHF rats. OPLS scores plots and permutation test of CON vs DOX group and DOX vs DOX + SXT (16 g/kg) group in positive ion (**A**, **C**) and negative ion (**B**, **D**); **E** heatmap of the vital changed metabolites in the CON, DOX and DOX + SXT(16 g/kg) groups; **F** Metabolic pathway analysis of differential metabolites with MetaboAnalyst; **G **Violin diagrams of 5 key metabolites in the phenylalanine metabolic pathway. ^#^*p* < 0.05, ^##^*p* < 0.01, ^###^*p* < 0.001 vs. the CON group; *p < 0.05 and **p < 0.01 vs. the DOX group

Orthogonal partial least squares discriminant analysis (OPLS-DA) of data from the CON and DOX groups on day 39 revealed a significant separation in both positive and negative ion modes. Permutation tests showed parameters of R2Y = 0.990 and Q2 = 0.909 in positive mode and R2Y = 0.998 and Q2 = 0.930 in negative mode, indicating reliable models without overfitting (Figs. 2A–D). Differential metabolites between CON and DOX groups were identified using variable importance in projection (VIP) values > 1.0 and *p*-values < 0.05, while differential metabolites between SXT and DOX groups were identified using values > 1.0 and *p*-values < 0.05. Based on log fold-change (FC) values, 81 potential biomarkers of SXT-mediated cardioprotection against DOX-induced toxicity were identified, with 42 metabolites significantly upregulated and 39 metabolites significantly downregulated compared to the DOX group (Table 1). Heatmap of the vital changed metabolites in the CON, DOX and SXT groups were presented in Fig. 2E.

**Table 1:** Summary of differential metabolites in urine

No	Rt (min)	VIP	Name	Formula	m/z	Adduct	Ion mode	CON vs. DOX	SXT vs. DOX
1	1.27	1.11	Galactarate	C_6_H_10_O_8_	209.0296	[M–H]^−^	ESI (–)	↑^*^	↑^***^
2	1.29	1.21	Glutamate	C_5_H_9_NO_4_	146.046	[M–H]^−^	ESI (–)	↑^**^	↑^*^
3	1.34	1.08	Threonate	C_4_H_8_O_5_	135.0293	[M–H]^−^	ESI (–)	↑^*^	↑^*^
4	1.37	1.71	Lactic acid	C_3_H_6_O_3_	89.0240	[M–H]^−^	ESI (–)	↑^***^	↑^***^
5	1.45	1.25	Threitol	C_4_H_10_O_4_	121.0499	[M–H]^−^	ESI (–)	↑^**^	↑^*^
6	1.56	1.54	Urea	CH_4_N_2_O	61.03970	[M + H]^+^	ESI (+)	↑^***^	↑^***^
7	1.77	1.13	alpha,alpha-Trehalose	C_12_H_22_O_11_	341.1086	[M–H]^−^	ESI (–)	↓^*^	↓^**^
8	1.77	1.61	Taurine	C_2_H_7_NO_3_S	126.0215	[M + H]^+^	ESI (+)	↑^***^	↑^***^
9	1.87	1.13	gamma-Glutamyl-beta-aminopropiononitrile	C_8_H_13_N_3_O_3_	217.1295	[M + NH_4_]^+^	ESI (+)	↓^*^	↓^**^
10	1.88	1.47	Imidazole lactate	C_6_H_8_N_2_O_3_	179.0443	[M + Na]^+^	ESI (+)	↓^***^	↓^*^
11	1.89	1.65	Pimelic acid	C_7_H_12_O_4_	159.0660	[M–H]^−^	ESI (–)	↑^***^	↑^**^
12	1.91	1.45	Prephenate	C_10_H_10_O_6_	261.0074	[M + Cl]^−^	ESI (–)	↑^***^	↑^**^
13	1.93	1.02	1F-beta-D-Fructosylsucrose	C_18_H_32_O_16_	539.1373	[M + Cl]^−^	ESI (–)	↓^*^	↓^***^
14	1.93	1.69	Cellopentaose	C_30_H_52_O_26_	827.2672	[M–H]^−^	ESI (–)	↓^***^	↓^**^
15	1.93	1.68	Stachyose	C_24_H_42_O_21_	665.2141	[M–H]^−^	ESI (–)	↓^***^	↓^***^
16	1.96	1.14	3-Oxalomalate	C_6_H_6_O_8_	240.9836	[M + Cl]^−^	ESI (–)	↑^**^	↑^*^
17	2.31	1.12	Cyromazine	C_6_H_10_N_6_	189.0866	[M + Na]^+^	ESI (+)	↓^*^	↓^**^
18	2.40	1.48	Pseudouridine	C_9_H_12_N_2_O_6_	267.0582	[M + Na]^+^	ESI (+)	↓^***^	↓^**^
19	2.41	1.47	Nicotinate	C_6_H_5_NO_2_	124.0389	[M + H]^+^	ESI (+)	↑^**^	↑^***^
20	2.71	1.07	Deoxycytidine	C_9_H_13_N_3_O_4_	228.0981	[M + H]^+^	ESI (+)	↓^*^	↓^*^
21	2.73	1.22	Serine-phosphoethanolamine	C_5_H_13_N_2_O_6_P	251.0403	[M + Na]^+^	ESI (+)	↑^*^	↑^***^
22	2.80	1.22	Oxalosuccinate	C_6_H_6_O_7_	224.9886	[M + Cl]^−^	ESI (–)	↑^**^	↑^***^
23	2.99	1.21	N-Acetyl-L-glutamate	C_7_H_11_NO_5_	190.0703	[M + H]^+^	ESI (+)	↑^*^	↑^*^
24	3.13	1.08	Pyridoxine	C_8_H_11_NO_3_	170.0809	[M + H]^+^	ESI (+)	↑^*^	↑^**^
25	3.18	1.44	dUMP	C_9_H_13_N_2_O_8_P	309.0577	[M + H]^+^	ESI (+)	↓^***^	↓^**^
26	3.25	1.56	2-Phosphoglycolate	C_2_H_5_O_6_P	156.9802	[M + H]^+^	ESI (+)	↑^***^	↑^**^
27	3.43	1.12	4-Aminobenzoate	C_7_H_7_NO_2_	136.0396	[M–H]^−^	ESI (–)	↑^*^	↑^**^
28	3.52	1.12	1-Methyladenosine	C_11_H_15_N_5_O_4_	282.1198	[M + H]^+^	ESI(+)	↓^*^	↓^***^
29	3.52	1.11	1-Methyladenine	C_6_H_7_N_5_	150.0766	[M + H]^+^	ESI (+)	↓^*^	↓^***^
30	4.44	1.64	Homovanillate	C_9_H_10_O_4_	205.0524	[M + Na]^+^	ESI (+)	↓^***^	↓^**^
31	4.67	1.23	L-Tyrosine	C_9_H_11_NO_3_	199.1071	[M + NH_4_]^+^	ESI (+)	↑^**^	↑^**^
32	4.86	1.45	Dopamine quinone	C_8_H_9_NO_2_	169.0966	[M + NH_4_]^+^	ESI (+)	↑^**^	↑^**^
33	4.89	1.28	2-Hydroxyadipate	C_6_H_10_O_5_	180.0878	[M + NH_4_]^+^	ESI (+)	↓^**^	↓^***^
34	4.89	1.16	alpha-Ribazole	C_14_H_18_N_2_O_4_	313.092	[M + Cl]^−^	ESI (–)	↓^**^	↓^**^
35	4.90	1.03	4-(2-Aminophenyl)-2,4-dioxobutanoate	C_10_H_9_NO_4_	206.0457	[M–H]^−^	ESI (–)	↓^*^	↓^*^
36	4.92	1.90	Xanthosine 5'-phosphate	C_10_H_13_N_4_O_9_P	382.0763	[M + NH_4_]^+^	ESI (+)	↓^***^	↓^***^
37	4.95	1.46	Xanthurenic acid	C_10_H_7_NO_4_	204.0300	[M–H]^−^	ESI (–)	↑^***^	↑^*^
38	4.96	1.50	L-Phenylalanine	C_9_H_11_NO_2_	164.0711	[M–H]^−^	ESI (–)	↓^***^	↓^*^
39	5.01	1.03	4-Pyridoxic acid	C_8_H_9_NO_4_	182.0460	[M–H]^−^	ESI (–)	↑^*^	↑^*^
40	5.02	1.48	Pantothenate	C_9_H_17_NO_5_	242.0995	[M + Na]^+^	ESI (+)	↑^**^	↑^**^
41	5.04	1.31	Nicotinurate	C_8_H_8_N_2_O_3_	179.0469	[M–H]^−^	ESI (–)	↓^**^	↓^*^
42	5.06	1.23	Hippurate	C_9_H_9_NO_3_	178.0511	[M–H]^−^	ESI (–)	↓^**^	↓^*^
43	5.08	1.78	Pantetheine 4'-phosphate	C_11_H_23_N_2_O_7_PS	359.0959	[M + H]^+^	ESI (+)	↓^***^	↓^**^
44	5.08	1.77	Phenylpyruvate	C_9_H_8_O_3_	165.0541	[M + H]^+^	ESI (+)	↓^***^	↓^***^
45	5.09	1.27	Acetyl phosphate	C_2_H_5_O_5_P	158.0270	[M + NH_4_]^+^	ESI (+)	↑^**^	↑^*^
46	5.24	1.89	S-Adenosyl-L-homocysteine	C_14_H_20_N_6_O_5_S	385.1227	[M + H]^+^	ESI (+)	↓^***^	↓^***^
47	5.38	1.15	3,4-Dihydroxybenzaldehyde	C_7_H_6_O_3_	172.9916	[M + Cl]^−^	ESI (–)	↓^**^	↓^***^
48	5.51	1.67	1-(5'-Phosphoribosyl)-5-formamido-4-imidazolecarboxamide	C_10_H_15_N_4_O_9_P	384.092	[M + NH_4_]^+^	ESI (+)	↓^***^	↓^***^
49	5.59	1.76	N,N-Dihydroxy-L-tyrosine	C_9_H_11_NO_5_	236.0551	[M + Na]^+^	ESI (+)	↓^***^	↓^**^
50	5.64	1.58	Tryptamine	C_10_H_12_N_2_	159.1019	[M–H]^−^	ESI (–)	↑^***^	↑^**^
51	5.66	1.68	5-Hydroxyconiferyl alcohol	C_10_H_12_O_4_	197.0804	[M + H]^+^	ESI (+)	↓^***^	↓^***^
52	5.67	1.53	3-(3-Hydroxyphenyl)propanoic acid	C_9_H_10_O_3_	165.0550	[M–H]^−^	ESI (–)	↑^***^	↑^***^
53	5.70	1.58	3-Methylsalicylate	C_8_H_8_O_3_	151.0394	[M–H]^−^	ESI (–)	↑^***^	↑^***^
54	5.73	1.06	Adrenaline	C_9_H_13_NO_3_	184.0964	[M + H]^+^	ESI (+)	↓^*^	↓^**^
55	5.85	1.64	S,S-Dimethyl-beta-propiothetin	C_5_H_10_O_2_S	135.0548	[M + H]^+^	ESI (+)	↓^***^	↓^**^
56	6.03	1.37	Homophenylalanine	C_10_H_13_NO_2_	197.1276	[M + NH_4_]^+^	ESI (+)	↑^**^	↑^*^
57	6.13	1.39	Quinaldic acid	C_10_H_7_NO_2_	174.0545	[M + H]^+^	ESI (+)	↑^**^	↑^**^
58	6.13	1.84	4-Hydroxyphenylacetate	C_8_H_8_O_3_	153.0541	[M + H]^+^	ESI (+)	↑^***^	↑^***^
59	6.14	1.19	(E)-Glutaconate	C_5_H_6_O_4_	148.0597	[M + NH_4_]^+^	ESI (+)	↓^**^	↓^***^
60	6.21	1.44	3-Indoleacrylate	C_11_H_9_NO_2_	186.0556	[M–H]^−^	ESI (–)	↑^***^	↑^***^
61	6.21	1.14	5-Methoxyindoleacetate	C_11_H_11_NO_3_	204.0661	[M–H]^−^	ESI (–)	↓^**^	↓^***^
62	6.27	1.38	Prostaglandin G2	C_20_H_32_O_6_	403.1958	[M + Cl]^−^	ESI (–)	↓^***^	↓^***^
63	6.27	1.53	Phenylacetylglutamine	C_13_H_16_N_2_O_4_	265.1176	[M + H]^+^	ESI (+)	↓^***^	↓^***^
64	6.34	1.07	Propionyladenylate	C_13_H_18_N_5_O_8_P	402.0800	[M–H]^−^	ESI (–)	↑^*^	↑^***^
65	6.36	1.20	3,4-Dihydroxymandelaldehyde	C_8_H_8_O_4_	169.0487	[M + H]^+^	ESI (+)	↑^*^	↑^***^
66	6.53	1.61	S-Adenosyl-L-methionine	C_15_H_22_N_6_O_5_S	433.1126	[M + Cl]^−^	ESI (–)	↓^***^	↓^***^
67	6.62	1.17	Harman	C_12_H_10_N_2_	200.1275	[M + NH_4_]^+^	ESI (+)	↑^*^	↑^*^
68	6.64	1.05	Deoxyguanosine	C_10_H_13_N_5_O_4_	266.0848	[M–H]^−^	ESI (–)	↓^*^	↓^**^
69	6.65	1.19	Phenylacetylglycine	C_10_H_11_NO_3_	194.0816	[M + H]^+^	ESI (+)	↓^*^	↓^*^
70	6.74	1.08	N-Acetyl-L-leucine	C_8_H_15_NO_3_	174.1117	[M + H]^+^	ESI (+)	↓^*^	↓^**^
71	6.76	1.41	Psilocybin	C_12_H_17_N_2_O_4_P	302.1235	[M + NH_4_]^+^	ESI (+)	↓^**^	↓^***^
72	6.92	1.43	4-Methyl-2-oxopentanoate	C_6_H_10_O_3_	131.0677	[M + H]^+^	ESI (+)	↓^**^	↓^**^
73	6.98	1.39	o-Toluate	C_8_H_8_O_2_	137.0590	[M + H]^+^	ESI (+)	↑^**^	↑^***^
74	7.15	1.51	Anthranilate	C_7_H_7_NO_2_	138.0549	[M + H]^+^	ESI (+)	↑^***^	↑^**^
75	7.24	1.34	Indole-3-acetaldehyde	C_10_H_9_NO	160.0749	[M + H]^+^	ESI (+)	↑^**^	↑^***^
76	7.24	1.05	Indolelactate	C_11_H_11_NO_3_	206.0810	[M + H]^+^	ESI (+)	↑^*^	↑^*^
77	7.43	1.71	Indole	C_8_H_7_N	118.0643	[M + H]^+^	ESI (+)	↑^***^	↑^***^
78	7.79	1.85	Indoleacetic acid	C_10_H_9_NO_2_	176.0703	[M + H]^+^	ESI (+)	↑^***^	↑^**^
79	9.47	1.09	Deoxycholic acid	C_24_H_40_O_4_	393.2912	[M + H]^+^	ESI (+)	↑^*^	↑^***^
80	11.93	1.23	Tridecanoic acid	C_13_H_26_O_2_	213.1854	[M–H]^−^	ESI (–)	↑^**^	↑^**^
81	12.41	1.30	2-Hydroxyestradiol	C_18_H_24_O_3_	311.1635	[M + Na]^+^	ESI (+)	↑^**^	↑^***^

^*^P < 0.05

^**^P < 0.01

^***^P < 0.001 vs. DOX group

Pathway analysis was conducted to elucidate the therapeutic mechanisms of SXT in DOX-induced CHF rats (Fig. 2F). Four top pathways were modulated after SXT treatment, including phenylalanine metabolism, phenylalanine, tyrosine and tryptophan biosynthesis, tryptophan metabolism, and arginine biosynthesis (Table S3). Fourteen key metabolites associated with these pathways were further analyzed. Compared to the CON group, the DOX group showed significantly increased urinary levels of 4-(2-aminophenyl)-2,4-dioxobutanoate, hippurate, phenylacetylglycine, 5-methoxyindoleacetate, phenylpyruvate, and phenylalanine, while the levels of indole-3-acetaldehyde, urea, anthranilate, indoleacetic acid, tryptamine, N-acetyl-L-glutamate, tyrosine, and glutamate were significantly decreased. SXT treatment largely normalized these metabolic disturbances (Figs. 2G and S3).

**Fig. 3:**
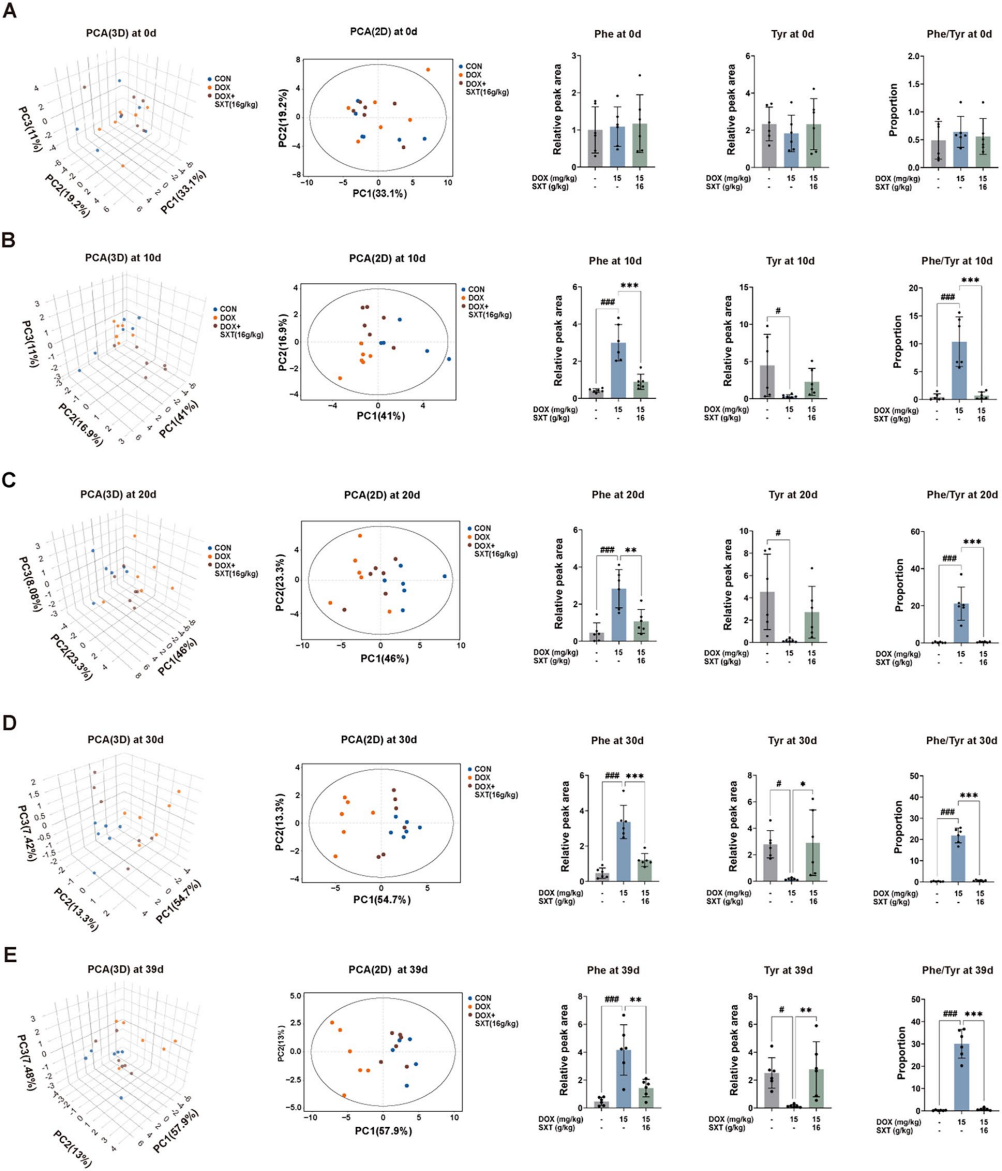
Urinary metabolites level change at different day. **A** 0 day; **B** 10 day; **C** 20 day; **D** 30 day; **E** 39 day. Phe: phenylalanine, Tyr: tyrosine. ^#^*p* < 0.05, ^###^*p* < 0.001 vs. the CON group; **p < 0.01, ***p < 0.001 vs. the DOX group

PCA revealed time-dependent changes (day 0, day 10, day 20, day 30 and day 39) in urine metabolites across all groups (Figs. 3A–E). The DOX group at day 30 showed the maximum shift along the PC1 axis (54.7%, largest variance explained) during 30- day-period, supporting the 30-day modeling period reported in the literature. In contrast, the SXT group showed a progressive reduction in metabolic distance from the CON group over the four time points (day 10, 20, 30, and 39), indicating that SXT administration can mitigate DOX-induced metabolic disturbances over time. This longitudinal metabolic change provided a comprehensive overview of the metabolic alterations associated with DOX-induced CHF progression and SXT treatment outcomes. Specifically, phenylalanine levels were significantly altered (Fig. 2G). As expected, it showed a sharp increase at the early perturbation phase following DOX administration (day 10, P < 0.001), and a rapid reduction after the SXT treatment (day 10, P < 0.001), with sustained decreases observed to experimental endpoint (P < 0.001 or P < 0.01). Similarly, after DOX intervention, tyrosine levels showed an opposite trend, with perturbations occurring at day 10 and persisting until the final time point (Figs. 3B–E). The phenylalanine/tyrosine ratio in urine samples, representing phenylalanine metabolism and excretion, showed a significant increase at day 10 that persisted until day 39 (Figs. 3B–E). These findings suggested that urinary phenylalanine excretion served as a compensatory mechanism for the substantially increased systemic phenylalanine exposure in CHF rats, although this effect was limited. Importantly, SXT treatment enhanced urinary phenylalanine excretion, restoring levels to those observed in the CON group.

### SXT alleviated gut microbiota disturbance in DOX-induced CHF rats

**Fig. 4:**
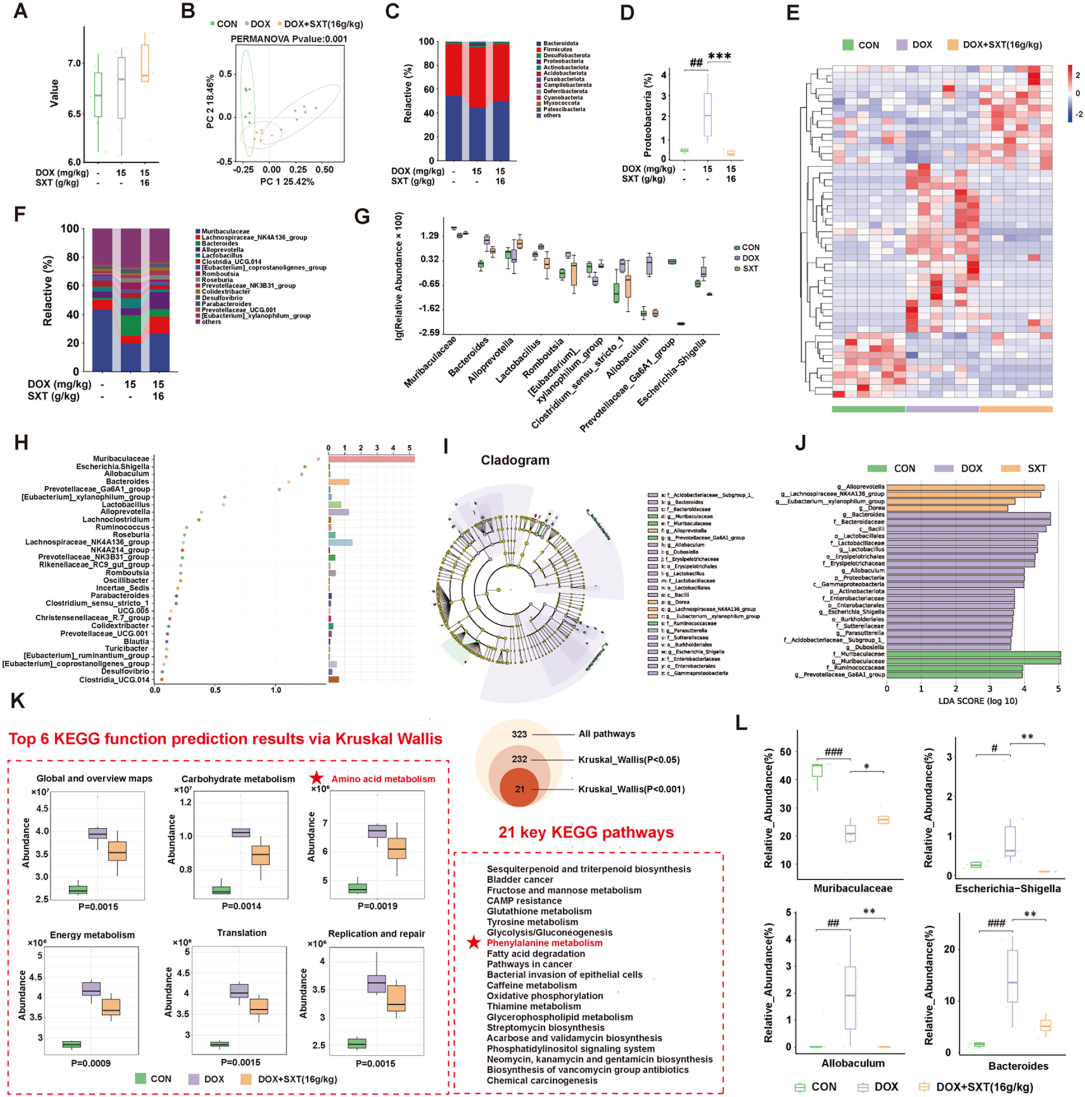
SXT alleviated gut microbiota disturbance in DOX-induced CHF rats. **A** Alpha-diversity analysis; **B** PCoA analysis of beta-diversity; **C** relative abundance of gut bacterial family; **D** the relative abundance of Proteobacteria; **E** Heatmap of differential *guts* at the genus level among CON, DOX and DOX + SXT(16 g/kg) groups; **F** relative abundance of *guts* bacterial genus; **G** the relative abundance of top 10 *guts* of beta-diversity; **H** Random forest model at the genus level; **I** Comparison of taxonomic abundances via LEfSe; **J** Histogram of linear discriminant analysis (LDA) of gut microbiota among different groups; **K** KEGG enrichment boxplot graph and 21 key pathways; **L** Top 4 genera with the most significant impacts on group discrimination. ^#^*p* < 0.05, ^##^*p* < 0.01, ^###^*p* < 0.001 vs. the CON group; *p < 0.05, **p < 0.01, ***p < 0.001 vs. the DOX group

Alpha-diversity analysis indicated no significant differences in diversity and richness among the samples from each group (Figs. 4A and S4A). Principal Coordinates Analysis (PCoA) based on the Bray–Curtis distance matrix revealed a distinct separation between the DOX and CON groups, while the SXT group showed an intermediate position, indicating that SXT treatment could partially alleviate DOX-induced gut microbiota dysbiosis (Fig. 4B). The clear separation among all three groups was further confirmed by Principal Component Analysis (PCA) (Figure S4B).

At the phylum level, the dominant bacteria in each group were *Bacteroidota*, *Firmicutes*, *Desulfobacterota*, and *Proteobacteria* (Fig. 4C). Compared to the CON group, the relative abundance of *Proteobacteria* was significantly increased in the DOX group (*p* < 0.01), which in the SXT group was significantly lower than that in the DOX group (*p* < 0.001) (Fig. 4D). Heatmap of differential guts among three groups was presented in Fig. 4E. At the genus level, *Muribaculaceae* was enriched in both the CON and SXT groups, while *Bacteroides* and *Escherichia-Shigella* were enriched in the DOX group (Figs. 4F and G).

Random Forest analysis (Fig. 4H) was performed to identify key bacterial taxa associated with treatment effects, with the differentially enriched taxa by LEfSe analysis (Figs. 4I and J). KEGG enrichment showed that phenylalanine metabolism was also involved in the top 21 key pathways (Fig. 4K). Results revealed that *Muribaculaceae*, *Escherichia-Shigella*, *Allobaculum*, and *Bacteroides*, *Prevotellaceae_GA6A1_group* were the top four genera with the most significant impacts on group discrimination. These taxa showed differential abundance patterns in comparisons between SXT, DOX, and CON groups, suggesting their potential roles in mediating the effects of DOX-induced cardiotoxicity and SXT treatment (Fig. 4L).

### SXT treatment altered phenylalanine metabolism in DOX-induced CHF rats by the integrative crosstalk of metabolomics and microbiome

**Fig. 5:**
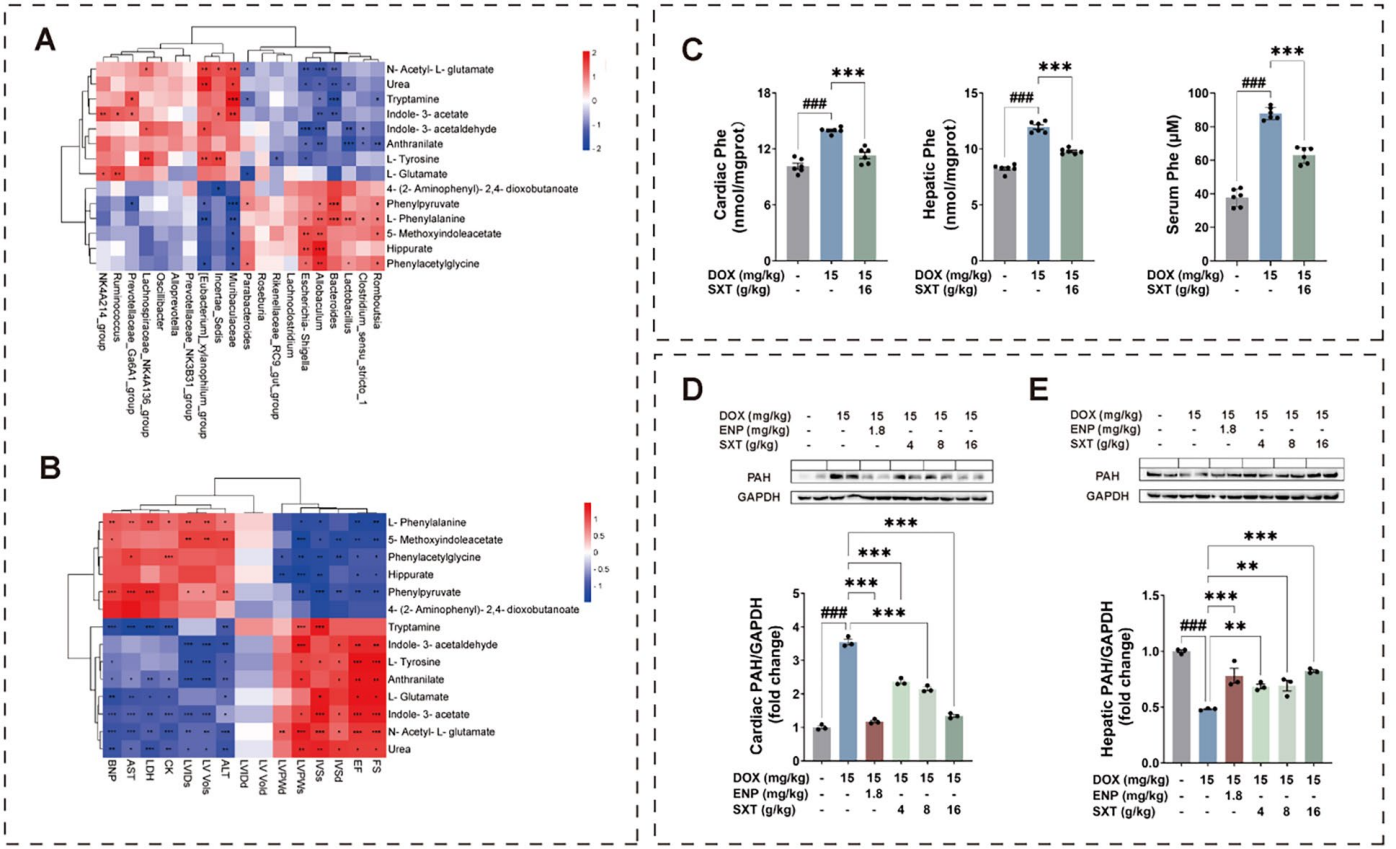
**A** correlation analysis of significantly changed metabolites and 20 significantly changed family; **B** correlation analysis of significantly changed metabolites and echocardiographic parameters; (**C**) phenylalanine levels in the bio-samples among the CON, DOX and SXT-H groups at day 39; western blotting and quantitative analysis of PAH in liver (**D**) and heart (**E**) tissues

The correlations between changes in 14 potential biomarkers and gut microbiomes were also investigated (Table 2, Fig. 5A). For instance, the abundance of *Muribaculaceae* was significantly negatively correlated with phenylalanine, phenylpyruvate, phenylacetylglycine and hippurate levels (*p* < 0.05), and positively correlated with N-acetyl-L-glutamate, urea, tryptamine and indoleacetic acid levels (*p* < 0.05). The abundance of *Escherichia-Shigella* was negatively correlated with tyrosine, indole-3-acetaldehyde, anthranilate, N-acetyl-L-glutamate and urea levels (*p* < 0.05). The abundance of *Bacteroides* was positively correlated with phenylalanine and phenylpyruvate (*p* < 0.001). Additionally, we analyzed correlations between metabolite changes and biochemical indices (Table 3, Fig. 5B). Most metabolites in the phenylalanine metabolic pathway, including phenylalanine, phenylpyruvate, phenylacetylglycine and hippurate, showed negative correlations with LVEF and LVFS (*p* < 0.05).

**Table 2:** Spearman correlation analysis between key differential metabolites and key differential gut microbes

	Indole-3-acetaldehyde	Phenylpyruvate	Indole-3-acetate	N-Acetyl-L-glutamate	Phenylacetylglycine	L-Tyrosine	Urea
Muribaculaceae	0.401	– 0.864***	0.699**	0.622**	– 0.498*	0.199	0.587*
Lachnospiraceae_NK4A136_group	0.509*	– 0.055	0.245	0.474*	– 0.319	0.692**	0.212
Bacteroides	– 0.368	0.818***	– 0.666**	– 0.633**	0.511*	– 0.437	– 0.616**
Alloprevotella	0.335	– 0.127	– 0.049	0.158	– 0.240	0.030	0.191
Lactobacillus	– 0.657**	0.385	– 0.404	– 0.385	0.356	– 0.461	– 0.488*
Romboutsia	– 0.544*	0.525*	– 0.404	– 0.393	0.494*	– 0.216	– 0.428
Roseburia	– 0.040	0.391	– 0.434	– 0.282	0.108	0.100	– 0.253
Prevotellaceae_NK3B31_group	0.071	– 0.020	– 0.174	– 0.001	– 0.100	– 0.139	0.077
Parabacteroides	– 0.125	0.496*	– 0.315	– 0.515*	0.486*	– 0.238	– 0.230
[Eubacterium]_xylanophilum_group	0.554*	– 0.482*	0.416	0.649**	– 0.550*	0.614**	0.680**
Ruminococcus	0.026	– 0.267	0.523*	0.179	– 0.094	0.362	0.053
Lachnoclostridium	– 0.284	0.321	– 0.443	– 0.294	– 0.038	– 0.385	– 0.284
Clostridium_sensu_stricto_1	– 0.476*	0.453	– 0.331	– 0.325	0.422	– 0.189	– 0.350
Rikenellaceae_RC9_gut_group	– 0.368	0.176	– 0.273	– 0.404	0.102	– 0.494*	– 0.205
Allobaculum	– 0.728***	0.550*	– 0.660**	– 0.739***	0.703**	– 0.463	– 0.534*
Prevotellaceae_Ga6A1_group	– 0.025	– 0.582*	0.485*	0.256	– 0.205	0.002	0.198
NK4A214_group	0.094	– 0.381	0.600**	0.162	– 0.022	0.428	0.323
Escherichia-Shigella	– 0.771***	0.455	– 0.379	– 0.719**	0.496*	– 0.569*	– 0.585*
Oscillibacter	0.424	0.069	0.096	0.267	– 0.362	0.443	0.131
Incertae_Sedis	0.408	– 0.354	0.474*	0.567*	– 0.317	0.653**	0.418

	Anthranilate	Tryptamine	L-Phenylalanine	Hippurate	5-Methoxyindoleacetate	4-(2-Aminophenyl)-2,4-dioxobutanoate	L-Glutamate
Muribaculaceae	0.410	0.812***	– 0.659**	– 0.494*	– 0.492*	– 0.354	0.288
Lachnospiraceae_NK4A136_group	0.273	0.082	– 0.236	– 0.377	– 0.389	– 0.232	0.404
Bacteroides	– 0.480*	– 0.752***	0.767***	0.410	0.410	0.470	– 0.527*
Alloprevotella	0.321	0.164	– 0.168	– 0.220	– 0.249	– 0.106	– 0.232
Lactobacillus	– 0.777***	– 0.302	0.616**	0.284	0.408	0.375	– 0.088
Romboutsia	– 0.602**	– 0.505*	0.534*	0.259	0.515*	0.179	– 0.282
Roseburia	– 0.166	– 0.335	0.271	0.170	0.112	– 0.112	0.271
Prevotellaceae_NK3B31_group	0.179	0.026	– 0.024	0.040	– 0.115	0.201	– 0.218
Parabacteroides	0.127	– 0.536*	0.207	0.463	0.337	0.263	– 0.569*
[Eubacterium]_xylanophilum_group	0.414	0.463	– 0.695**	– 0.459	– 0.441	– 0.414	0.455
Ruminococcus	0.290	0.238	– 0.160	0.073	– 0.172	0.046	0.620**
Lachnoclostridium	– 0.410	– 0.259	0.404	0.222	0.245	0.257	0.121
Clostridium_sensu_stricto_1	– 0.542*	– 0.453	0.484*	0.179	0.441	0.220	– 0.317
Rikenellaceae_RC9_gut_group	– 0.358	– 0.042	0.205	0.195	0.391	– 0.170	– 0.166
Allobaculum	– 0.645**	– 0.489*	0.671**	0.777***	0.600**	0.334	– 0.066
Prevotellaceae_Ga6A1_group	0.180	0.546*	– 0.427	– 0.159	– 0.169	– 0.230	0.370
NK4A214_group	0.451	0.201	– 0.457	– 0.001	– 0.240	– 0.146	0.552*
Escherichia-Shigella	– 0.548*	– 0.389	0.505*	0.614**	0.624**	0.319	0.034
Oscillibacter	0.218	0.129	– 0.191	– 0.296	– 0.191	– 0.123	0.236
Incertae_Sedis	0.172	0.393	– 0.362	– 0.331	– 0.311	– 0.536*	0.432

^*^p < 0.05

^**^p < 0.01

^***^p < 0.001

**Table 3:** Spearman correlation analysis between key differential metabolites and key pharmacodynamic index

	Indole-3-acetaldehyde	Phenylpyruvate	Indole-3-acetate	N-Acetyl-L-glutamate	Phenylacetylglycine	L-Tyrosine	Urea
ALT	– 0.688^**^	0.649^**^	– 0.488^*^	– 0.717^**^	0.455	– 0.571^*^	– 0.676^**^
AST	– 0.327	0.911^**^	– 0.744^**^	– 0.763^**^	0.531^*^	– 0.432	– 0.552^*^
BNP	– 0.410	0.818^**^	– 0.802^**^	– 0.779^**^	0.453	– 0.480^*^	– 0.678^**^
CK	– 0.432	0.789^**^	– 0.732^**^	– 0.771^**^	0.457	– 0.391	– 0.591^**^
LDH	– 0.377	0.907^**^	– 0.787^**^	– 0.659^**^	0.445	– 0.354	– 0.692^**^
IVSd	0.558	– 0.561	0.596	0.514	– 0.635	0.483	0.549
IVSs	0.474	– 0.792	0.897	0.744	– 0.678	0.516	0.618
LVIDd	– 0.327	– 0.145	– 0.376	– 0.047	– 0.115	– 0.388	– 0.172
LVIDs	– 0.721	0.496	– 0.817	– 0.621	0.415	– 0.714	– 0.544
LVPWd	0.322	– 0.215	0.430	0.565	– 0.555	0.435	0.330
LVPWs	0.739	– 0.652	0.666	0.865	– 0.695	0.559	0.605
LVEF	0.642	– 0.635	0.848	0.745	– 0.527	0.765	0.537
LVFS	0.642	– 0.635	0.848	0.745	– 0.527	0.765	0.537
LV Vold	– 0.327	– 0.145	– 0.376	– 0.047	– 0.115	– 0.388	– 0.172
LV Vols	– 0.721	0.496	– 0.817	– 0.621	0.415	– 0.714	– 0.544

	Anthranilate	Tryptamine	L-Phenylalanine	Hippurate	5-Methoxyindoleacetate	4-(2-Aminophenyl)-2,4-dioxobutanoate	L-Glutamate
ALT	– 0.633^**^	– 0.643^**^	0.517^*^	0.323	0	0.123	– 0.488^*^
AST	– 0.496^*^	– 0.899^**^	0	0.420	0.412	0.424	– 0.595^**^
BNP	– 0.558^*^	– 0.771^**^	0.614^**^	0.410	0.494^*^	0.373	– 0.645^**^
CK	– 0.542^*^	– 0.783^**^	0.544^*^	0.366	0.395	0.269	– 0.569^*^
LDH	– 0.606^**^	– 0.878^**^	0	0.224	0.383	0.342	– 0.577^*^
IVSd	0.523	0.451	– 0.415	– 0.443	– 0.687	– 0.310	0.414
IVSs	0.440	0.727	– 0.544	– 0.613	– 0.587	– 0.343	0.514
LVIDd	– 0.371	0.278	0.236	0.017	0.111	– 0.157	– 0.167
LVIDs	– 0.777	– 0.279	0.639	0.460	0.616	0.223	– 0.427
LVPWd	0.370	0.144	– 0.444	– 0.629	– 0.383	– 0.091	0.026
LVPWs	0.589	0.503	– 0.553	– 0.709	– 0.817	– 0.199	0.232
LVEF	0.711	0.458	– 0.642	– 0.493	– 0.664	– 0.260	0.534
LVFS	0.711	0.458	– 0.642	– 0.493	– 0.664	– 0.260	0.534
LV Vold	– 0.371	0.278	0.236	0.017	0.111	– 0.157	– 0.167
LV Vols	– 0.777	– 0.279	0.639	0.460	0.616	0.223	– 0.427

^*^p < 0.05

^**^p < 0.01

^***^p < 0.001

In the metabolic profile, phenylalanine levels exhibited a significant elevation in hepatic and cardiac tissues concurrent with plasma accumulation in DOX-induced CHF rats, while pharmacological intervention with SXT demonstrated marked attenuation of this dysregulation, restoring phenylalanine concentrations approaching to the normal values from the CON group at the final time point (day 39, Fig. 5C). Therefore, western blotting analysis was conducted to investigate the tissue-specific expression of phenylalanine hydroxylase (PAH), the key rate-limiting enzyme for phenylalanine to tyrosine metabolism. It was revealed that compared to the CON group, the DOX group showed a significantly decreased liver PAH expression (*p* < 0.001) and a significantly increased ectopic heart PAH expression (*p* < 0.001). Compared to the DOX group, the SXT groups showed a significantly increased liver PAH expression (*p* < 0.01) and a significantly decreased ectopic heart PAH expression (*p* < 0.001) (Figs. 5D and E). Notably, PAH concentration could not be determined in the heart tissue from the NOR group. These results indicated that DOX inhibited liver PAH expression and induced ectopic PAH expression in the heart, while SXT significantly mitigated this effect.

Based on these results, we constructed a co-expression network of key metabolites, genera, absorbed compounds, and echocardiographic and biochemical parameters showing differential expression following SXT administration in DOX-induced CHF rats using Cytoscape 3.9.0 software (Figure S5, |r|> 0.5). The network involved 14 metabolites in the four pathways, 15 gut microbiota genera into four clusters, 18 blood-absorbed compounds after SXT administration, and two echocardiographic parameters and five biochemical parameters indicating cardiac and hepatic functions. The co-expression networks revealed that phenylalanine metabolism had strong associations with the presented gut microbiota genera, absorbed compounds, and echocardiographic and biochemical parameters. Additionally, the visualization demonstrated strong correlations between tyrosine and tryptophan biosynthesis and the aforementioned indices.

### SXT treatment improved DOX-induced CHF by reversing abnormal phenylalanine metabolism

**Fig. 6:**
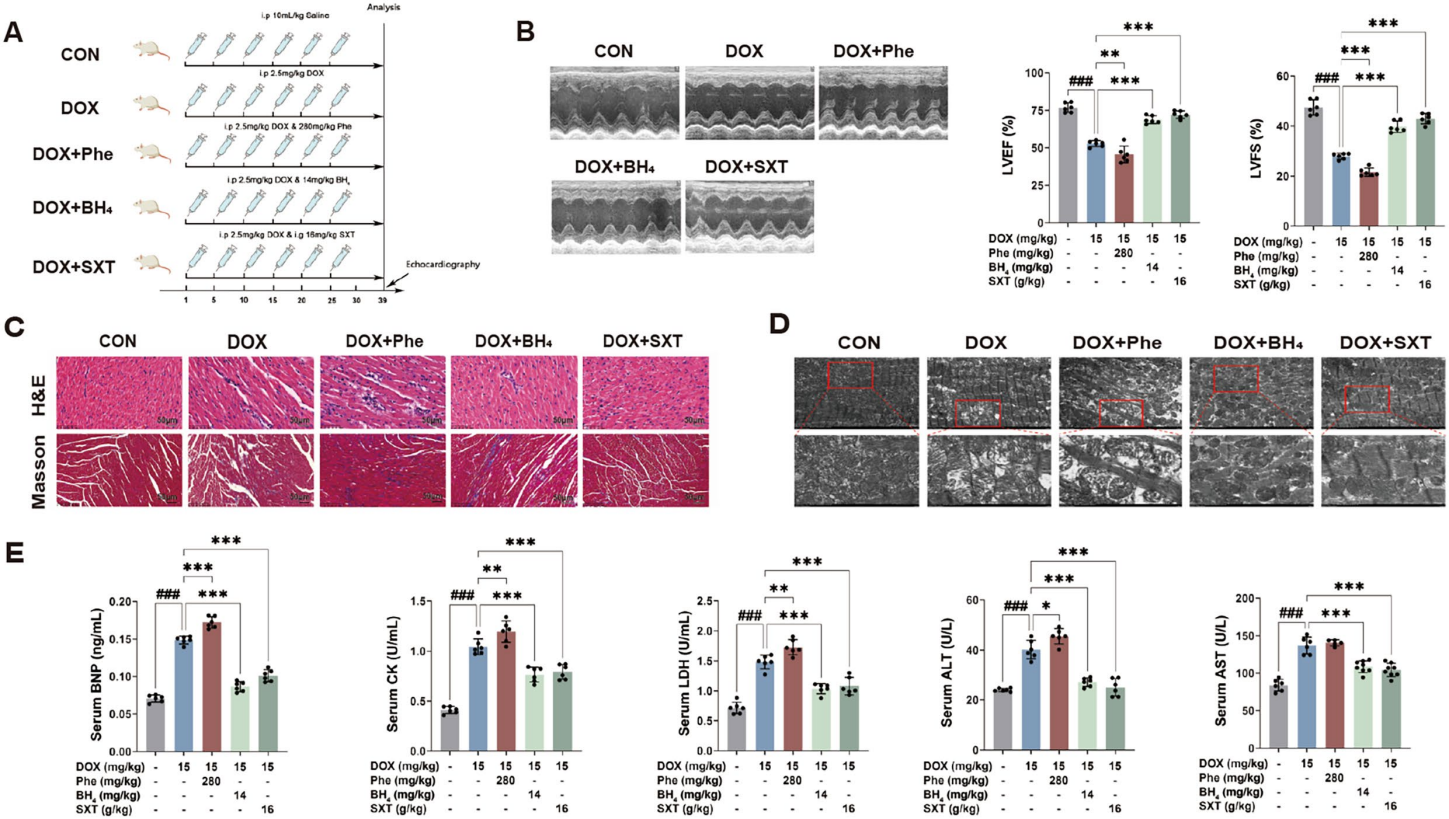
Phenylalanine exacerbates DOX-induced CHF which is ameliorated by SXT treatment. **A** Schematic protocol of the experiment II; **B** representative sequential measurements of echocardiograms, LVEF % and LVFS %; **C** representative sections of hearts stained with H&E, and masson's trichrome for fibrosis detection (blue), scale bars 50 µm; **D** representative TEM serum; **E** BNP, CK, LDH, ALT, and AST levels in serum. ^###^*p* < 0.001 vs. the CON group; *p < 0.05, **p < 0.01 and ***p < 0.001 vs. the DOX group

Following the study design shown in Fig. 6A, we investigated whether phenylalanine metabolism played a critical role in DOX-mediated cardiotoxicity. The phenylalanine degradation pathway cofactor BH_4_ was employed as a positive control. Cardiac function indicators were also evaluated in the rats given DOX with BH_4_ (DOX + BH_4_ group) and the rats administered with DOX and SXT (DOX + SXT group). Echocardiography showed lower LVEF and LVFS in the DOX + Phe group compared to the DOX group (P < 0.001), while BH_4_ and SXT addition partially alleviated DOX-induced cardiac dysfunction (Fig. 6B). H&E and Masson staining demonstrated that phenylalanine exacerbated DOX-induced cardiac injury and collagen accumulation, while BH_4_ supplementation or SXT intervention ameliorated these effects (Fig. 6C). Electron microscopy revealed that DOX induced mitochondrial shrinkage, increased membrane density, and marked reduction and shortening of cristae with focal vacuolization. These effects were more pronounced with phenylalanine intervention but reversed by BH_4_ or SXT treatment (Fig. 6D). Phenylalanine co-administration largely promoted DOX-induced increases in serum levels of BNP, CK, LDH, ALT and AST (*p* < 0.05), while BH_4_ supplementation or SXT treatment significantly inhibited the elevation of these markers in DOX-induced CHF rats (*p* < 0.001) (Fig. 6E).

**Fig. 7:**
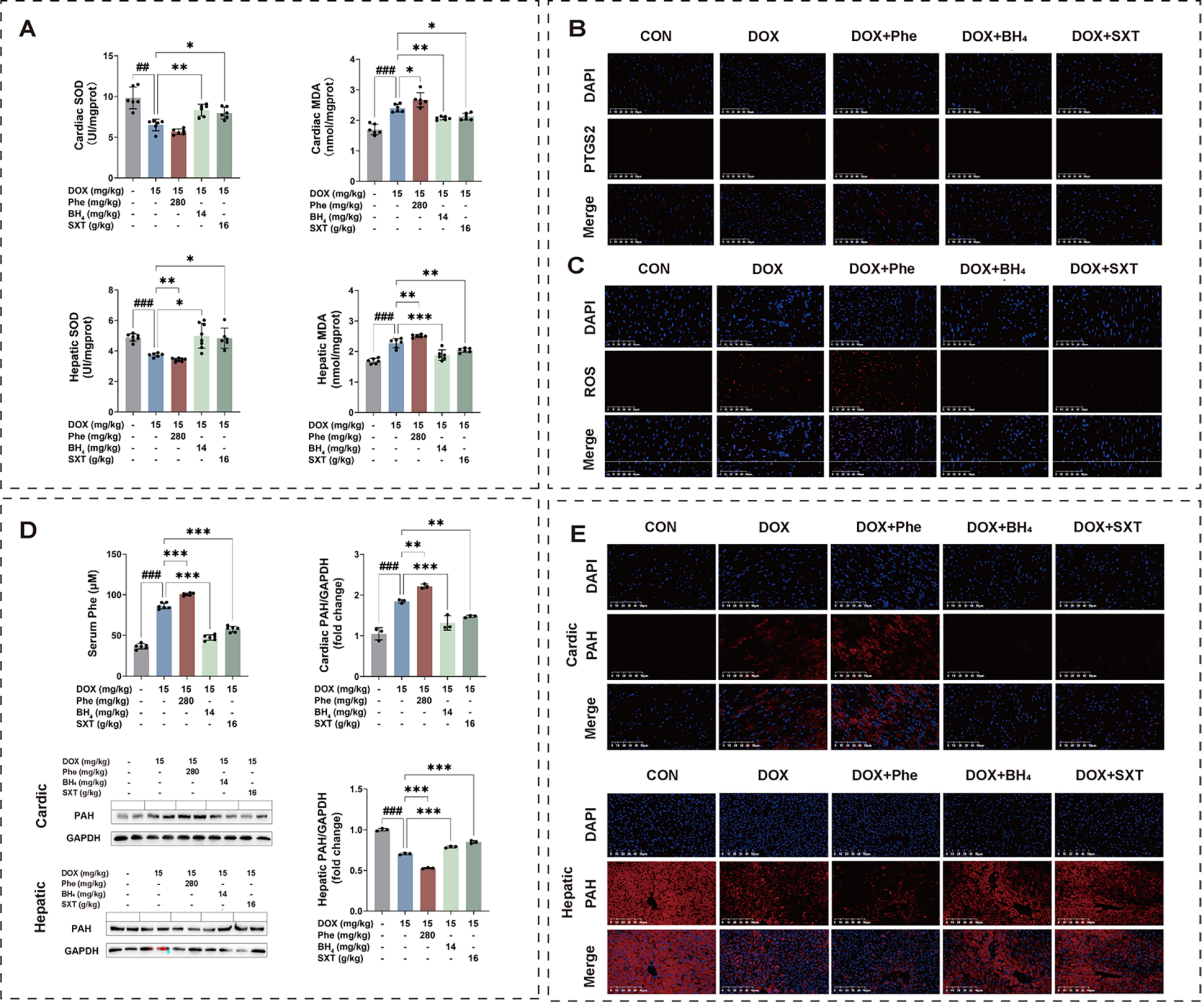
SXT alleviated DOX-induced oxidative stress and restored phenylalanine metabolism. **A** levels of SOD and MDA in heart and liver tissue; immunofluorescence images of PTGS2 (**B**) and ROS (**C**); **D** serum phenylalanine levels, and quantitative analysis of PAH in in liver and heart tissues; **E** immunofluorescence images of PAH in in liver and heart tissues. ^###^*p* < 0.001 vs. the CON group; *p < 0.05, **p < 0.01 and ***p < 0.001 vs. the DOX group

Compared to the DOX group, phenylalanine administration exacerbated DOX-induced cardiac oxidative stress by the pronounced suppression of hepatic and cardiac SOD activity alongside marked MDA elevation (*P* < 0.01 or *P* < 0.001), when compared with those of the DOX group (Fig. 7A). Moreover, phenylalanine supplementation also further ​amplified the oxidative damages, as evidenced by upregulating ROS production and PTGS2 expression in the heart, while co-treatment with SXT or BH_4_ effectively attenuated the pathological alterations by DOX (Figs. 7B and C). However, the dysregulation by DOX was effectively attenuated by BH_4_ or SXT pretreatment, suggesting the capacity of SXT to restore the oxidative stress balance.

To determine if BH_4_ supplementation or SXT treatment improved phenylalanine metabolism, we measured phenylalanine concentrations in all rats. DOX significantly increased serum phenylalanine concentration (86.1433 μM) (*p* < 0.001), which was further elevated after phenylalanine co-administration (100.715 μM) (*p* < 0.001). BH_4_ (14 mg/kg) supplementation and SXT (16 g/kg) treatment reduced serum phenylalanine concentrations to 46.9791 μM and 57.8214 μM, respectively (*p* < 0.001) compared to the DOX group (Fig. 7D). We thus hypothesized that SXT treatment would improve phenylalanine catabolism by enhancing PAH activity, similar to BH_4_ supplementation. To test this hypothesis, we conducted western blotting and immunofluorescence analyses to examine PAH expression in liver and heart tissues. As expected, DOX intervention substantially lowered PAH expression in liver tissue, while significantly raising PAH expression in heart tissue. After BH_4_ supplementation, PAH concentration increased in liver tissue and decreased in heart tissue. Similarly, SXT treatment induced an increase in liver tissue and a decrease in heart tissue for PAH concentration (*p* < 0.001) (Figs. 7D and E). The results showed that phenylalanine treatment further exacerbated DOX-induced decreases in liver PAH expression and increases in ectopic heart PAH expression in CHF rats, while both BH_4_ and SXT significantly inhibited abnormal PAH expression in the liver and heart of DOX-induced CHF rats.

Furthermore, exogenous phenylalanine administration exacerbated DOX-induced cardiotoxicity in H9C2 cells, as evidenced by decreased cell viability, suppressed SOD activity, and elevated CK, LDH, and MDA levels (Figure S6). These results corroborated the preceding observations, demonstrating that phenylalanine pretreatment potentiated DOX-mediated cellular damage.

### ***Absorbed compounds from SXT strongly bind to PAH to maintain stable interaction between PAH and BH***_***4***_

UHPLC-QTOF/MS-based identification of absorbed constituents in the liver revealed 14 compounds, including timosaponin A-III, cimigenol xyloside, timosaponin B-II, anemarrhenasaponin I, neomangiferin, astragaloside IV, calycosin-7-O-β-D-glucoside, ononin, timosaponin A-II, daidzein, calycosin, formononetin, isoferulic acid, and azelaic acid (Table S4, Figure S7). Docking status of PAH protein with those compounds was shown in Figure S8, as well as DOX and BH_4_. Further molecular docking analysis confirmed that anemarrhenasaponin I, timosaponin B-II, calycosin-7-O-β-D-glucoside, timosaponin A-III and neomangiferin showed a higher binding affinity to the PAH protein, with binding energies of − 11.1, − 11.3, -9.9, − 12.2 and − 10.2 kcal/mol, respectively, which were also lower than BH_4_ (− 7.1 kcal/mol) (Table 4).

**Table 4:** Docking status of active ingredients and BH_4_ with PAH protein

No	The binding ingredients	CAS No	Energy (kcal/mol)
1	Timosaponin A-III	41059-79-4	– 12.2
2	Cimigenol xyloside	27994-11-2	– 11.4
3	Timosaponin B-II	136656-07-0	– 11.3
4	Anemarrhenasaponin I	163047-21-0	– 11.1
5	Neomangiferin	64809-67-2	– 10.2
6	Astragaloside IV	84687-43-4	– 10.1
7	Calycosin-7-O-β-D-glucoside	20633-67-4	– 9.9
8	Ononin	486-62-4	– 9.7
9	DOX	23214-92-8	– 9.5
10	Timosaponin A-II	117210-12-5	– 9.4
11	Daidzein	486-66-8	– 9.0
12	Calycosin	20575-57-9	– 8.5
13	Formononetin	485-72-3	– 8.3
14	Isoferulic acid	537-73-5	– 7.1
15	BH_4_	17528-72-2	– 7.1
16	Azelaic acid	123-99-9	– 5.3

Results for the binding sites showed that DOX could form hydrogen bonds with PAH through glu-381, ser-349 and ser-350, exerting a high pharmacodynamic activity. BH_4_ could form hydrogen bonds with PAH through his-264, glu-286, leu-249, and glu-330, which differed from those of DOX. As for the tested bioactive compounds, timosaponin B-II formed hydrogen bonds with PAH through his-264, ser-251, leu-249, gly-352, ser-350, and met-276; timosaponin A-III through ser-350, leu-249, and glu-286; anemarrhenasaponin I through his-264 and ser-251; calycosin-7-O-β-D-glucoside through his-264, leu-249, glu-286 and glu-353; and neomangiferin through ser-350, met-276 and thr-278. Some of the binding sites for these five compounds were the same as those of DOX. These molecular docking results indicate that the above five compounds could strongly bind to PAH, potentially inhibiting DOX binding and preventing reduced expression of PAH by maintaining stable interactions between PAH and BH_4_.

### Neomangiferinn, anemarrhenasaponin I, timosaponin B II, and timosaponin A III from SXT could inhibit the DOX-induced cell injury

In the AML12 cells, non-cytoxic concentration of the test compounds were identified as follows: for the timosaponins, like timosaponin A-II and timosaponin A-III, 0.1–0.5 µM; for the phenolic acids and flavonoids, including BH_4_, azelaic acid, daidzein, isoferulic acid, formononetin, calycosin, ononin, and calycosin-7-O-β-D-glucoside, 5–20 µM; and for the ​saponins and their derivatives, including astragaloside IV, timosaponin B-II, neomangiferin, anemarrhenasaponin I and cimigenol xyloside, 5–20 µM. Cell viability test showed that all compounds from SXT could exhibit a dose-dependent increase in DOX-induced AML12 cell viability (Figures S9A-S9C). Furtherly, phenylalanine levels in AML12 cells showed significant increases after DOX intervention (*P* < 0.001), which could be markedly decreased after BH_4_ (10 μM) intervention (*P* < 0.01). Meanwhile, it was found that timosaponin B-II (5, 10, and 20 μM), neomangiferin (5, 10, and 20 μM), anemarrhenasaponin I (5, 10, and 20 μM), and timosaponin AIII (0.1, 0.2, and 0.5 μM) could significantly reduce the increased phenylalanine levels in AML12 cells caused by DOX (Figures S9D–F). The four compounds were visualized in the Venn chart (Figure S9G).

### Neomangiferin from SXT mitigated DOX-induced AML12 cells injury and oxidative stress by enhancing the homeostatic binding to PAH

**Fig. 8:**
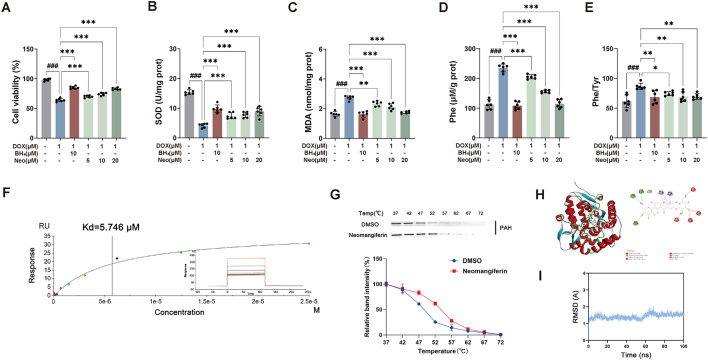
Neomangiferin mitigated DOX-treated AML12 cell injured and oxidative stress by strongly bind to PAH. (**A**) Cell viability; (**B**) SOD; (**C**) MDA; (**D**)Phe level; (**E**) Phe/Tyr ratio; (**F**) SPR analysis results of neomangiferin and PAH; (**G**) western blotting results of CETSA assay samples (n = 3); (**H**) docking status of PAH with neomangiferin; (**I**) root means square deviation (RMSD) result for PAH protein. Neo: Neomangiferin. ^###^*p* < 0.001 vs. the CON group; *p < 0.05, **p < 0.01 and ***p < 0.001 vs. the DOX group

We evaluated the impact of DOX on AML12 cells by assessing cell viability and oxidative stress markers. DOX treatment induced in a significant reduction in cell viability and SOD activity, concomitant with an increase in MDA levels (*p* < 0.001). Neomangiferin alleviated the DOX-induced reduction in cell viability and oxidative stress in a dose-dependent manner (*p* < 0.01, *p* < 0.001) (Figs. 8A–C). Consistent with previous findings, DOX treatment significantly suppressed phenylalanine metabolism (p < 0.001), an effect that was ameliorated by Neomangiferin in a dose-dependent manner (*p* < 0.05, *p* < 0.01) (Figs. 8D and E).

Surface plasmon resonance (SPR) experiments showed that anemarrhenasaponin I, timosaponin B-II, timosaponin A III, and neomangiferin had comparable slower dissociation constant (Kd) values (< 10 μM), in contrast to a higher value for BH_4_ (10.65 μM) (Figure S10, Table S5). Interestingly, the phenylalanine/tyrosine ratio in AML12 cell, representing PAH activity, showed a significant increase only in the neomangiferin group (Figure S11). To explore the potential target and molecular mechanisms of neomangiferin, CETSA experiments and molecular dynamics simulation were performed. CETSA results showed that compared with the control group, the PAH protein treated with neomangiferin had a better thermal stability (Fig. 8F), with a binding Kd value of 5.881 nM by SPR analyse (Fig. 6G). As the docking score value of neomangiferin was − 10.2 kcal/mol (Fig. 6H), the molecular dynamics simulation was further carried out for these docked complexes to understand their conformational their stability and binding energy. Root means square deviation (RMSD) results showed the protein stabilized at about 70 ns (Fig. 6I). Besides, Root Means Square Fluctuation (RMSF), Radius of Gyration (RG), Solvent Accessible Surface Area (SASA) and hydrogen bonds analyses were separately calculated and visually analyzed, indicating the neomangiferin and PAH showed strong interactions (Figure S12). In conclusion, these results provided robust evidence for the high stability of neomangiferin-PAH complexes.

## Discussion

This study demonstrated SXT’s preventive effects on DOX-induced CHF by systematically assessment of echocardiography test, biochemical markers, oxidative stress indicators, and pathological observations. A combined omics strategy was conducted to provide new insights into the role of phenylalanine metabolism in DOX-induced CHF, with potentially important therapeutic implications.

A longitudinal urinary metabolomics approach combined with microbiome analysis was used to investigate the systemic metabolome and gut microbiome in CHF rats and the therapeutic value of SXT, along with histopathological evaluation and biochemical assays. Additionally, the integration analysis revealed that dysregulated phenylalanine metabolism secondary to ectopic hepatic phenylalanine hydroxylase (PAH) protein expression emerged as an early-onset feature of DOX-induced CHF, which was verified in vivo and in vitro. This led us to hypothesize and then confirm that compounds from SXT could correct the ectopic PAH expression. These findings highlight SXT’s protective effects and hepatic PAH-mediated phenylalanine catabolism as a potential therapeutic target for DOX-induced CHF.

The selection of the AML12 hepatocyte line to validate the regulatory effect of neomangiferin on PAH is based on our proposed systemic mechanism, in which the cardioprotective action of SXT is partially mediated through a “hepatic-metabolic-cardiac crosstalk” talk. As the central organ for phenylalanine metabolism, the liver expresses PAH as the key rate-limiting enzyme controlling systemic and organ-specific phenylalanine levels. The hepatic phenylalanine metabolism was directly modulated by SXT and its active component via the upregulated PAH expression in hepatocytes, and recovered the disturbed phenylalanine homeostasis. SXT's upregulation of PAH in hepatocytes thus establishes critical upstream evidence linking phenylalanine balance restoration to cardiac functional improvement. To elucidate hepatic-metabolic-cardiac crosstalk, we further evaluated phenylalanine's impact on DOX-treated H9C2 cardiomyocytes. Exogenous phenylalanine amplified DOX toxicity, evidenced by the reducing cell viability, diminishing SOD activity, and elevating levels of CK, LDH, and MDA. This finding was consistent with our in vivo observations in rats. Although the direct effects of SXT compounds on cardiomyocytes were not examined here, our integrated in vivo and in vitro evidence defines a previously unrecognized role for phenylalanine metabolism in DOX-induced cardiotoxicity, providing novel mechanistic insight into the cardioprotective pathway of SXT. While this study focused on SXT's systemic effects, our integrated approach reveals a novel metabolic-cardiac axis where phenylalanine dysregulation constitutes a critical pathway in DOX cardiotoxicity.

Urine metabolomics was employed in this study, as the advantages of easy and repeatable collection, and ability to reflect imbalances across all biochemical pathways within the body [7, 32]. The metabolomic data unexpectedly revealed a dysregulation of phenylalanine and tryptophan metabolism, and the elevated phenylalanine levels were confirmed to increase in plasma, liver and heart samples by the established quantitative method, indicating the effectiveness of using urinary samples for analysis. In addition, this study also verified an increased level of phenylpyruvic acid and phenylacetylglycine, which were involved in pro-hypertrophic, pro-fibrotic, and pro-inflammatory effects on cardiomyocytes and cardiac fibroblasts [24, 27]. Hippuric acid, a gut-derived protein-bound uremic toxin with an incident cardiovascular disease risk, also showed an increased abundance in the DOX group. All the mentioned compounds’ abundance was decreased after SXT intervention. The urinary tryptamine and indole-3-acetate exposure decreased in the DOX group, potentially attenuating indicators of inflammation in macrophages. 5-methoxyindoleacetic acid, another tryptophan metabolite, showed increased urinary levels in the DOX group, suggesting activation of oxidative injury [29]. Anthranilic acid and 4-(2-aminophenyl)-2,4-dioxobutanoic acid, metabolites from other branches of the tryptophan pathway, exhibited significant disturbances in urine, further indicating inflammatory responses and microbiota homeostasis disruption [23]. Those compounds abundance returned to normal level after SXT intervention, confirming their therapeutic contribution to CHF.

Based on this evidence, we proposed that gut microbiota were involved as a potential mechanism mediating SXT’s effect. In this study, several members of the microbial families *Muribaculaceae* and *Lachnospiraceae* were significantly altered in the DOX induced CHF rats. Briefly, *Muribaculaceae* showed a high abundance in the CON group, decreased in the DOX group, but exhibited a significant recovery trend in the SXT group (*p* < 0.05), consistent with research in other animal models with HF [33]. Conversely, *Lachnospiraceae* exhibited an increase in the DOX group but showed improved recovery after SXT treatment, as *Lachnospiraceae* was shown to increase the risk of HF [36]. The further integrated microbiome and metabolome analysis revealed that *Muribaculaceae* and *Bacteroidetes* were related to various significantly altered metabolites, including phenylalanine, phenylpyruvate, and phenylacetylglycine in phenylalanine metabolism. These findings highlighted phenylalanine metabolism modulation as a potential therapeutic strategy for DOX-induced CHF.

Elevated phenylalanine has been observed in CHF patients, with prognostic values [4, 5, 45]. The patients with high phenylalanine have even been identified a unique metabolic phenotype with the adverse outcomes in acute decompensated HF [3]. However, those studies were lack of the causes of elevated phenylalanine levels, as well as the potential phenylalanine targeted interventions such as dietary restrictions or drug treatments. Notably, higher concentrations of phenylalanine indicate elevated protein turnover and stress response to reduced cardiac output [6]. This led us to hypothesize that high systemic phenylalanine exposure could trigger cardiac dysfunction following DOX administration. To confirm phenylalanine’s role in exacerbating cardiac abnormalities, rats that received DOX were fed with phenylalanine concurrently. Given that phenylalanine accumulation in HF patients indicates BH_4_ depletion, and that increasing myocardial BH_4_ levels through supplementation can prevent or reverse LV dysfunction [1], BH_4_ was employed as the positive control in experiment II. As expected, oral phenylalanine administration increased serum phenylalanine levels by 16.92%, while BH_4_ supplementation and SXT treatment decreased serum phenylalanine levels by 83.37% and 74.18%, respectively, compared to the DOX group. Interestingly, PAH was primarily expressed in the liver, whose decreased expression could cause hepatic phenylalanine accumulation, leading to high phenylalanine exposure in the serum and heart. Results in this study exhibited a markedly reduced hepatic PAH expression and an increased cardiac PAH expression in both DOX and DOX + phenylalanine (DOX + Phe) groups, while hepatic PAH expression significantly increased and cardiac PAH expression reduced in both DOX + BH_4_ and DOX + SXT groups. These data demonstrated that the development of cardiac ectopic phenylalanine catabolism was caused by DOX, which could be prevented by BH_4_ supplementation or SXT treatment. Observations from structural, functional, and molecular alterations in the rats further verified this hypothesis.

Importantly, DOX has been proven to generate excessive ROS and oxygen radicals, as cardiac function heavily relies on mitochondrial oxidative metabolism [40]. This investigation also identified a significant ROS accumulation in rat cardiomyocytes after DOX stimulation, which further increased with phenylalanine intervention, indicating that it exacerbated oxidative stress in DOX-induced CHF. Concurrently, phenylalanine addition significantly reduced cardiac and hepatic SOD levels while increasing cardiac and hepatic MDA levels. TEM results showed that compared to the normal group, the number of swollen mitochondria in rat cardiomyocytes significantly decreased in both DOX and DOX + Phe groups. Both BH_4_ and SXT interventions effectively reduced ROS levels in rat cardiomyocytes, increased cardiac and hepatic SOD levels, decreased cardiac and hepatic MDA levels, and mitigated mitochondrial swelling in cardiomyocytes. These results suggested that SXT could detoxify or repair damages caused by DOX and phenylalanine by maintaining the balance of intracellular oxidants and antioxidants, similar to the effects of BH_4_.

PAH deficiency leads to elevated blood phenylalanine levels, often caused by PAH protein instability and misfolding with loss of function [13]. Importantly, PAH structural changes are sensitive to the affinity for the substrate phenylalanine and cofactor BH_4_ [12], suggesting that PAH expression is influenced by molecules that can bind to PAH. This observation was confirmed by previous reports that PAH deficiency did not adapt liver BH_4_ concentrations, which could be partially corrected by pharmacological doses of BH_4_ [8]. Our current study proved that timosaponin B-II, neomangiferinn, anemarrhenasaponin I and timosaponin A-III, the absorbed compounds in the liver of DOX-induced CHF rats, could inhibit the DOX-induced AML-12 cell injury and increased phenylalanine level. Those compounds were thus conducted in the subsequent molecular docking, molecular dynamics simulation, CETSA and SPR analysis. It was revealed that neomangiferin showed a significantly higher binding affinity for the key target PAH. Moreover, the enzyme activity assay confirmed that neomangiferin could significantly increase PAH activity. We thus speculated that hepatic PAH was a possible therapeutic target, whose activity could be increased by neomangiferin via its strong binding to exert protective effects.

Our study had several strengths. Firstly, this was the first study to systematically investigate the pharmacological effects of SXT on DOX-induced CHF through integrated omics profiling, establishing a novel mechanistic framework for cardioprotection. Secondly, the longitudinal metabolic analysis coupled with ectopic cardiac phenylalanine catabolism assessment revealed that dysregulated phenylalanine metabolism not only served as a diagnostic biomarker but also actively contributed to cardiac dysfunction progression. Thirdly, data obtained from computer simulation and experimental verification demonstrated that neomangiferin could stabilize PAH-BH4 interaction through competitive binding with PAH, effectively attenuating DOX-induced PAH inhibition while preserving enzymatic activity.

Several limitations of this study should be noted. In the exploratory correlation analyses integrating metabolomic, microbiome, and pharmacodynamic data, statistical correction for multiple comparisons was not applied. This decision was made to allow the detection of all potential biomarker signals and cross-domain interactions, which is appropriate for hypothesis-generating analyses. However, the absence of multiple-comparison adjustment increases the possibility of false-positive correlations. Therefore, the identified associations should be interpreted with caution. While these findings provide valuable leads for future mechanistic investigation, their biological relevance and any causal links will require validation in independent cohorts or through targeted experimental approaches, such as gain- or loss-of-function studies or direct metabolite intervention.

In addition, several important limitations should also be taken into consideration. While serum, hepatic and cardiac phenylalanine levels were profiled, comprehensive quantification of BH_4_ and phenylalanine downstream metabolites should be assayed to elucidate the full metabolic cascade. The cardioprotective effects of neomangiferin should be validated in DOX-exposed PAH-deficient hepatic models to determine tissue-specific therapeutic windows and potential compensatory mechanisms.

## Conclusion

In conclusion, our integrated metabolomics and microbiome analysis demonstrated that SXT effectively attenuated DOX-induced CHF in rats through a novel mechanistic pathway involving phenylalanine metabolism dysregulation. Our findings confirmed elevated phenylalanine levels directly contribute to DOX induced cardiac dysfunction via ectopic catabolism, revealing ectopic phenylalanine catabolism as a critical mediator of CHF progression. SXT showed early protective effects, and neomangiferin enhanced PAH activity by stabilizing its interaction with BH_4_, thereby blocking DOX binding and preserving enzymatic function. Our results supported SXT as a promising candidate for of DOX-induced CHF prevention, with hepatic PAH as a possible therapeutic target.

## Declaration of generative Al and Al-assisted technologes in the writing process

Not applicable.

## Supplementary Information

Supplementary Material 1.

## Data Availability

The raw datasets used and analyzed during the study are available on request to the senior author (Feng Zhang, [fengzhang@smmu.edu.cn] [mailto:fengzhang@smmu.edu.cn]).

## Abbreviations

ALT: Alanine aminotransferase
AST: Aspartate aminotransferase
BCA: Bicinchoninic acid
BNP: Brain natriuretic peptide
CETSA: Cellular thermal shift assay
CHF: Chronic heart failure
CK: Creatine kinase
CMC-Na: Carboxymethyl cellulose sodium
DAPI: 4′,6-Diamidino-2-phenylindole
DCFH-DA: 2′,7′-Dichlorofluorescein diacetate
DOX: Doxorubicin
ECG: Echocardiographic
ELISA: Enzyme-linked immunosorbent assay
ENP: Enalapril
FC: Fold change
H&E: Hematoxylin and eosin
HPLC: High performance liquid chromatography
IDA: Information-dependent acquisition
LC: Liquid chromatography
LDH: Lactate dehydrogenase
LVEF: Left ventricular ejection fraction
LVFS: Left ventricular fractional shortening
MDA: Malondialdehyde
MS: Mass spectrometry
NIH: National institutes of health
OPLS-DA: Orthogonal partial least squares discriminant analysis
OTUs: Operational taxonomic units
PAH: Phenylalanine hydroxylase
PCA: Principal component analysis
PCoA: Principal coordinates analysis
PTGS2: Prostaglandin-endoperoxide synthase 2
PVDF: Polyvinylidene fluoride
QC: Quality control
ROS: Reactive oxygen species
SD: Sprague Dawley
SDS-PAGE: Sodium dodecyl sulfate–polyacrylamide gel electrophoresis
SOD: Superoxide dismutase
SPR: Surface plasmon resonance
SXT: Sheng-Xian-Tang
TCMs: Traditional Chinese medicines
TOF: Time of flight
TUNEL: Transferase-mediated dideoxyuridine triphosphate-biotin nick end labeling
UPLC: Ultra performance liquid chromatography
VIP: Variable importance in projection
