# Supplementary material for: Therapeutic potential of Sheng-Xian-Tang in doxorubicin-induced chronic heart failure by regulation of phenylalanine metabolism disruption
Source: Chin Med. 2026 Jan 12;21:29. doi: 10.1186/s13020-025-01316-6 (PMC12794274; doi:10.1186/s13020-025-01316-6)
Supplement: Supplementary file 1 — Supplementary Material 1. [file 13020_2025_1316_MOESM1_ESM.docx]

**Supporting information**

*Original Article*

**Therapeutic potential of Sheng-Xian-Tang in doxorubicin-induced chronic heart failure by regulation of phenylalanine metabolism disruption**

Tao Pang^1#^, Chao Wang^1,2#^, Guangyang Jiao^1,2^, Xiangcheng Fan^3,4^, Doudou Huang^5^, Zhimin Long^6^, Mengqing Xiao^6^, Lianna Sun^5*^, Wansheng Chen^1,2,7*^, Feng Zhang^1,7*^

*1 Department of Pharmacy, Changzheng Hospital, Naval Medical University (Second Military Medical University), Shanghai 200003, China.*

*2 The SATCM Key Laboratory for New Resources & Quality Evaluation of Chinese Medicine, Institute of Chinese Materia Medica, Shanghai University of Traditional Chinese Medicine, Shanghai 201203, China*

*3 Tongde Hospital of Zhejiang Province Afflicted to Zhejiang Chinese Medical University (Tongde Hospital of Zhejiang Province), Hangzhou, Zhejiang, 310014, China*

*4 Zhejiang Academy of Traditional Chinese Medicine, Hangzhou, Zhejiang, 310014, China*

*5 School of Pharmacy, Shanghai University of Traditional Chinese Medicine, Shanghai 201203, China*

*6 AB SCEIX, Shanghai 200050, China*

*7 Shanghai Key Laboratory for Pharmaceutical Metabolite Research, Naval Medical University (Second Military Medica University), Shanghai, 200433, China*

^#^These authors contributed equally to this work.

**1. Methods**

*1.1 Detection of absorbable compounds from SXT in the blood, heart and liver samples from DOX-induced CHF rats by UHPLC-Q-TOF/MS analysis*

Rat serum (300 μL) were vortexed for 3 min with 1.2 mL of 0.1% formic acid in methanol (containing 200 ng/mL internal standard warfarin). Liver samples (100 mg) were homogenized with 1 mL of 70% methanol by tissuelyser. After centrifugation for 3000 r/min for 10 min at 4 °C, the supernatant was collected. The mixture was centrifuged at 13000 rpm for 3 min at 4 °C. The prepared serum, heart or liver samples were collected respectively, dried under vacuum condition, and the residues were redissolved in 120 μL of 80% methanol and vortexed for 3 min. Those samples were centrifuged at 13000 rpm for 10 min at 4 °C before UHPLC-Q-TOF/MS analysis (Figure S3).

UHPLC-Q-TOF/MS analysis of the absorbable compounds from SXT in the blood and liver samples were conducted using an Agilent 1290 ultra-performance liquid chromatography system with an Agilent 6530 high resolution mass spectrometer (Agilent Technologies) as the previously published work (Jiao et al., 2024). Briefly, separation was performed on an Agilent Infinity Lab Poroshell 120 PFP column (2.1 mm ×150 mm, 2.7 μm). Mobile phases were composed of 0.1% formic acid in water (phase A) and acetonitrile (phase B), using a gradient elution: 0–10–25–35–38–40 min, 3–25–45–85–95–95% phase B. The flow rate and the injection volume were set to 0.35 mL/min and 3 μL, respectively. The temperatures for the autosampler and the column were set at 4°C and 35°C, respectively. Mass data for acquisition: ion source, dual AJS ESI; gas temperature, 310 °C; gas flow, 8.0 L/min; nebulizer, 45 psi; sheath gas temp, 325 °C; sheath gas flow, 10 L/min; nozzle voltage, 500 V; vcap, 4.0 kV in positive mode and 3.5 kV in negative mode; MS1 and MS2 scan range, 100−1700 m/z; MS2 fragmentor, 130 V; MS2 collision energy, 30 eV.

The liver tissue was identified in total 14 compounds, including timosaponin A-III, cimigenol xyloside, timosaponin B-II, anemarrhenasaponin I, neomangiferin, astragaloside IV, calycosin-7-O-β-D-glucoside, ononin, timosaponin A-II, daidzein, calycosin, formononetin, isoferulic acid, and azelaic acid, which were all detected in the serum (Table S3).

*1.2Molecular docking and Molecular dynamics simulation*

Molecular docking simulations were performed to predict the binding affinity and conformation between core targets and key active ingredients (AutoDock Vina 1.1.2). The pdb format files of PAH were obtained from the RCSB Protein Data Bank (https://www.rcsb.org), and crystallographic waters and heterogeneous atoms were removed using PyMOL (https://pymol.org) before loading the files into AutoDockTools. In AutoDockTools, atom types were assigned and Gasteiger charges were added to the receptor pdb format files before saving them in pdbqt format as docking receptors. The structure files of active ingredients were downloaded in sdf format from the PubChem database (https://pubchem.ncbi.nlm.nih.gov) and converted to pdb format using Open Babel 2.4.1 (https://sourceforge.net/projects/openbabel/files/openbabel/). Ligand pdb files were then loaded into AutoDock Tools where atom types were assigned and Gasteiger charges were added, before being saved in pdbqt format to prepare docking ligands. AutoDock Vina was applied to compute binding affinities between ligands and receptors for affinity assessment, and PYMOL was employed to molecular docking result visualization.

Molecular dynamics simulations were conducted using Gromacs 2022.3 software. Following system minimization and relaxation, a 100 ns simulation was performed with three independent runs to improve statistical robustness. Trajectory coordinates were recorded at intervals of 100 ps. Post-simulation, trajectory corrections were applied, followed by stability and interaction analyses, including Root Means Square Deviation (RMSD), Root Means Square Fluctuation (RMSF), Radius of Gyration (Rg), Solvent Accessible Surface Area (SASA) and hydrogen bond analysis.

*1.3 Evaluation of the effect of phenylalanine on doxorubicin-induced H9C2 cells.*

H9C2 cells were seeded in 96-well plates at a density of 5 × 10⁴ cells per well (in 100 µL medium) and allowed to adhere for 12 h. The cells were then treated for 24 h with either DOX (1 µM) alone or DOX (1 µM) in combination with Phe (18.5 µM; a concentration derived from the dosing conversion ratio used in prior rat experiments). An untreated group served as the control. Cell viability was determined by measuring the optical density at 450 nm using a microplate reader. The levels of CK, LDH, SOD, and MDA were quantified using commercial assay kits.

**2. Supplementary figures**


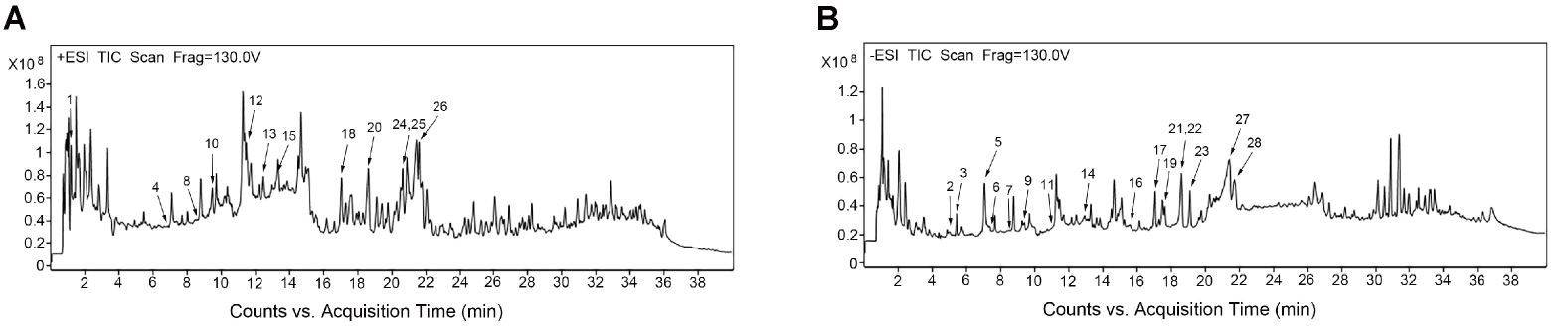


**Figure S1.** The total ion chromatography of SXT in positive (A) and negative (B) modes by UHPLC-Q-TOF/MS.

Compounds 1-28 were listed in table S3.


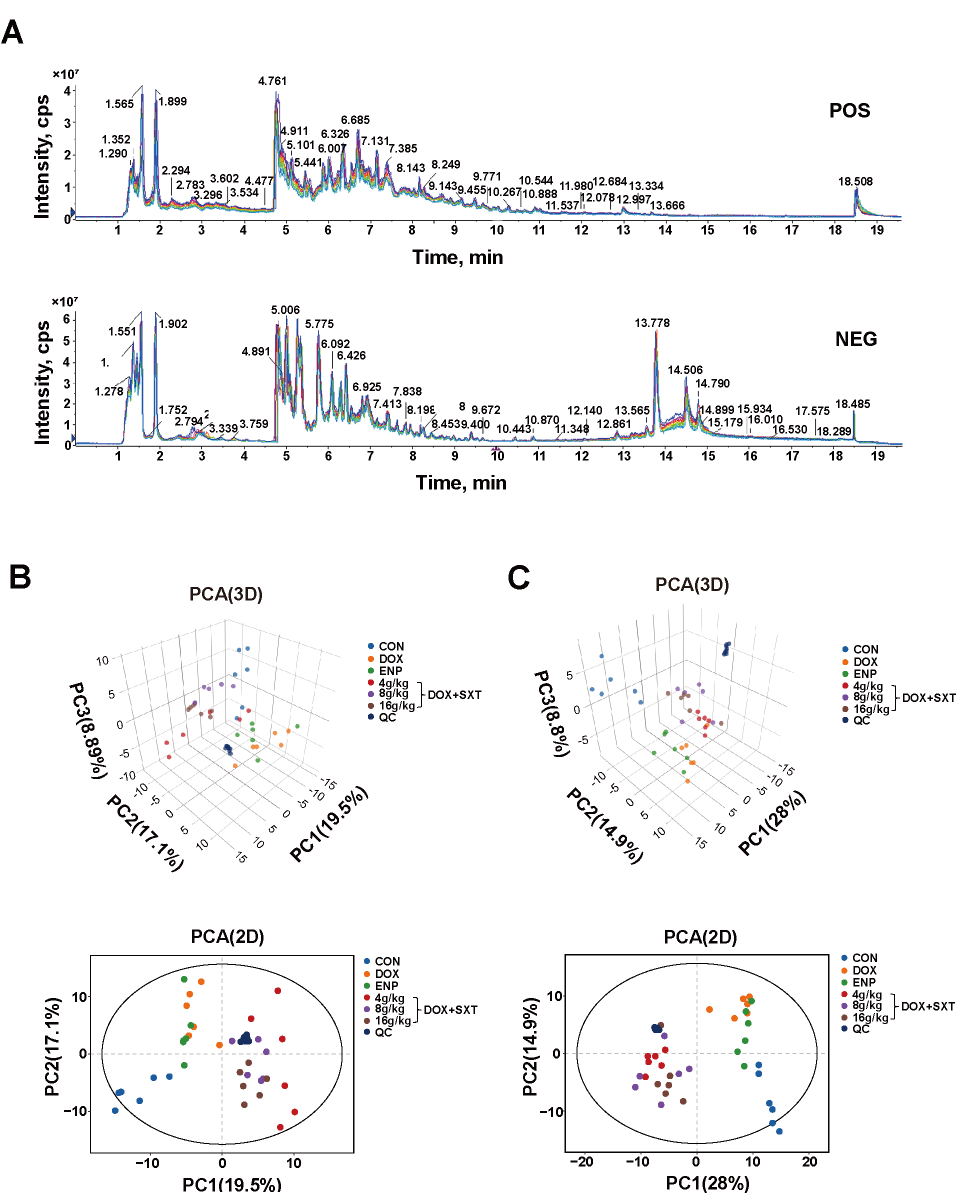


**Figure S2.** Profiling and multivariate statistical analysis.

Total ion chromatograms (TIC) of the rat urine obtained in positive **(A)** and negative **(B)** modes; PCA score plot of all samples and QCs in positive **(B)** and negative **(C)** modes; PCA score plot among CON, DOX and DOX+SXT (16g/kg) groups at 1d **(D)**, 10d **(E)**, 20d **(F)**, and 30d **(G)** in positive.


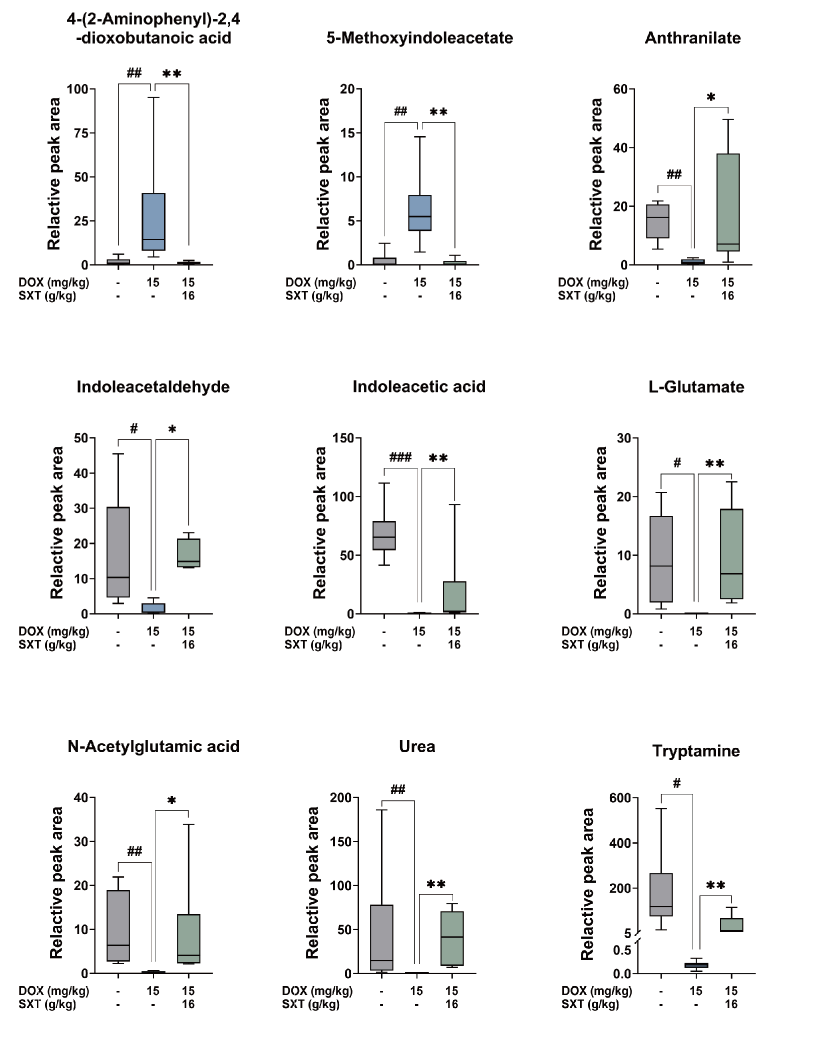


**Figure S3.** Concentrations of the 9 key metabolites among the control (CON), the model (DOX) and the high-dose of SXT (16 g/kg) groups.

^#^*p* < 0.05, ^##^*p* < 0.01, ^###^*p* < 0.001 vs. the CON group; **p* < 0.05, ***p* < 0.01, ****p* < 0.001 vs. the DOX group.

**
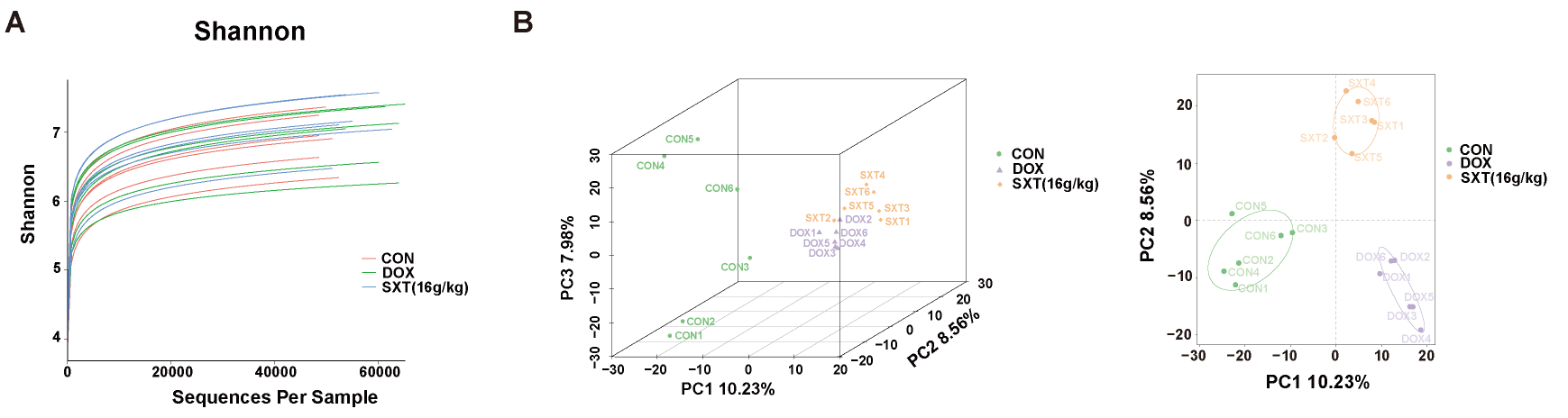
**

**Figure S4. (A)**Shannon; **(B)** graph of PCA score among CON, DOX, and SXT (16g/kg).


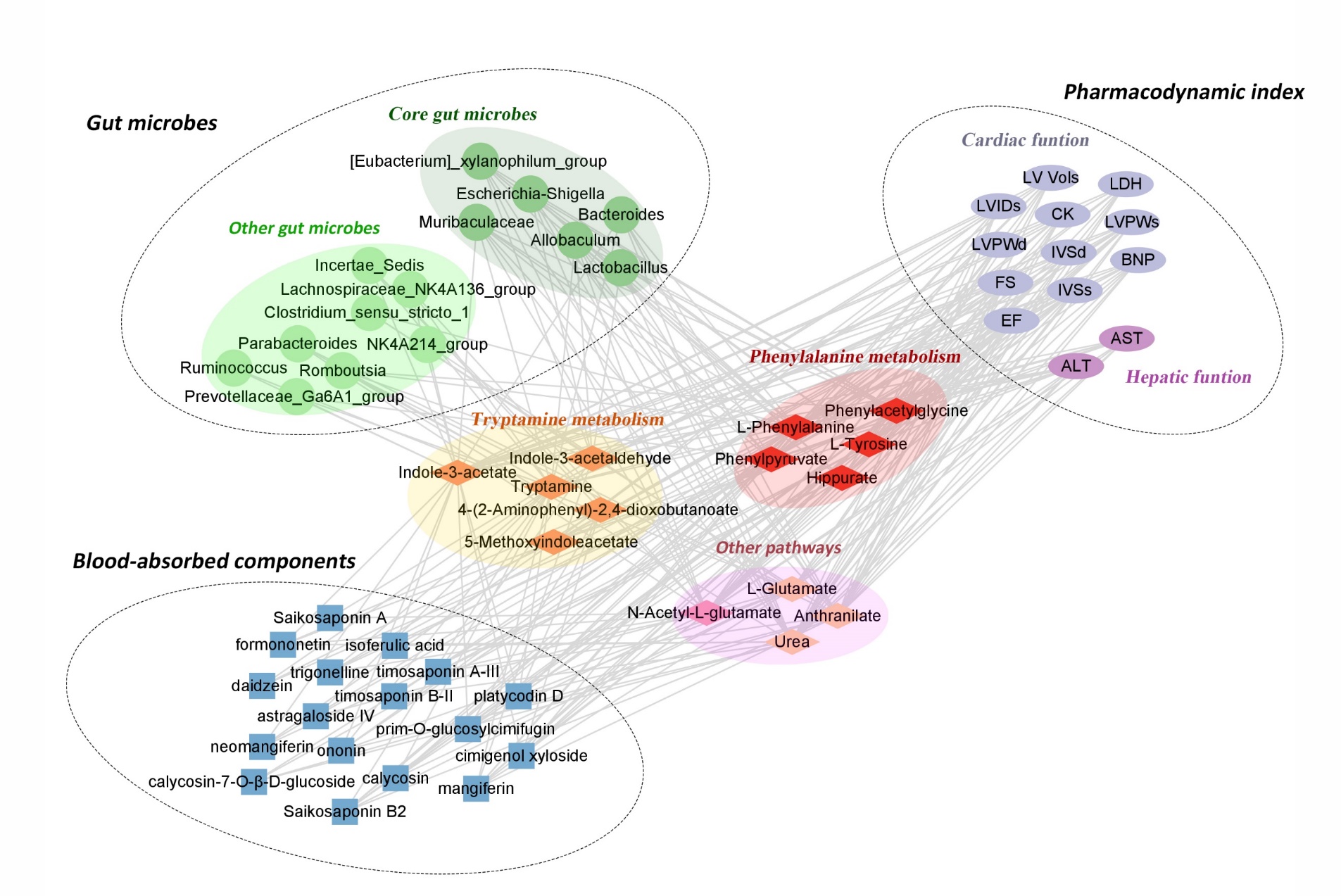


**Figure S5.** Co-expression networks between key metabolites, key genera, key absorbed compounds, and key echocardiographic and biochemical parameters that show differential expression following SXT administration in DOX induced CHF rats.

Diamonds represent different metabolites, circles represent different genera, square represent different absorbed compounds from SXT, and ellipse represent different echocardiographic and biochemical parameters.


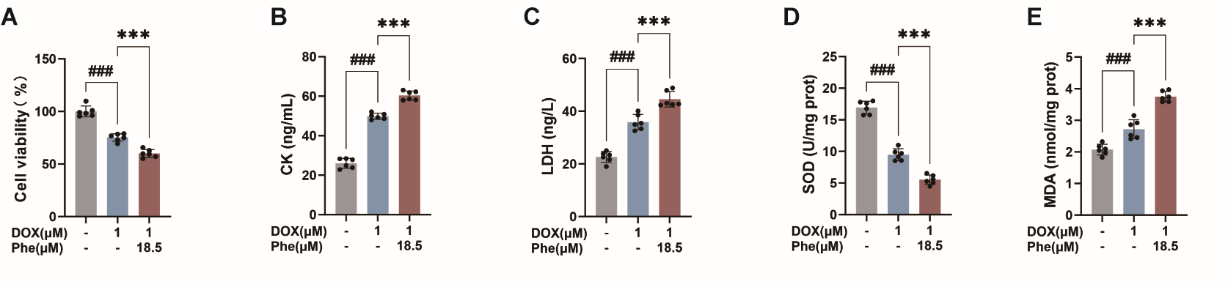


**Figure S6.** Phenylalanine aggravated DOX-induced injury in H9C2 cells. (**A**) Cell viability; (**B**) CK; (C) LDH; (D) SOD; (E) MDA. Phe: phenylalanine, ^###^*p*<0.001 vs. the CON group; ****p* < 0.001 vs. the DOX group.

**
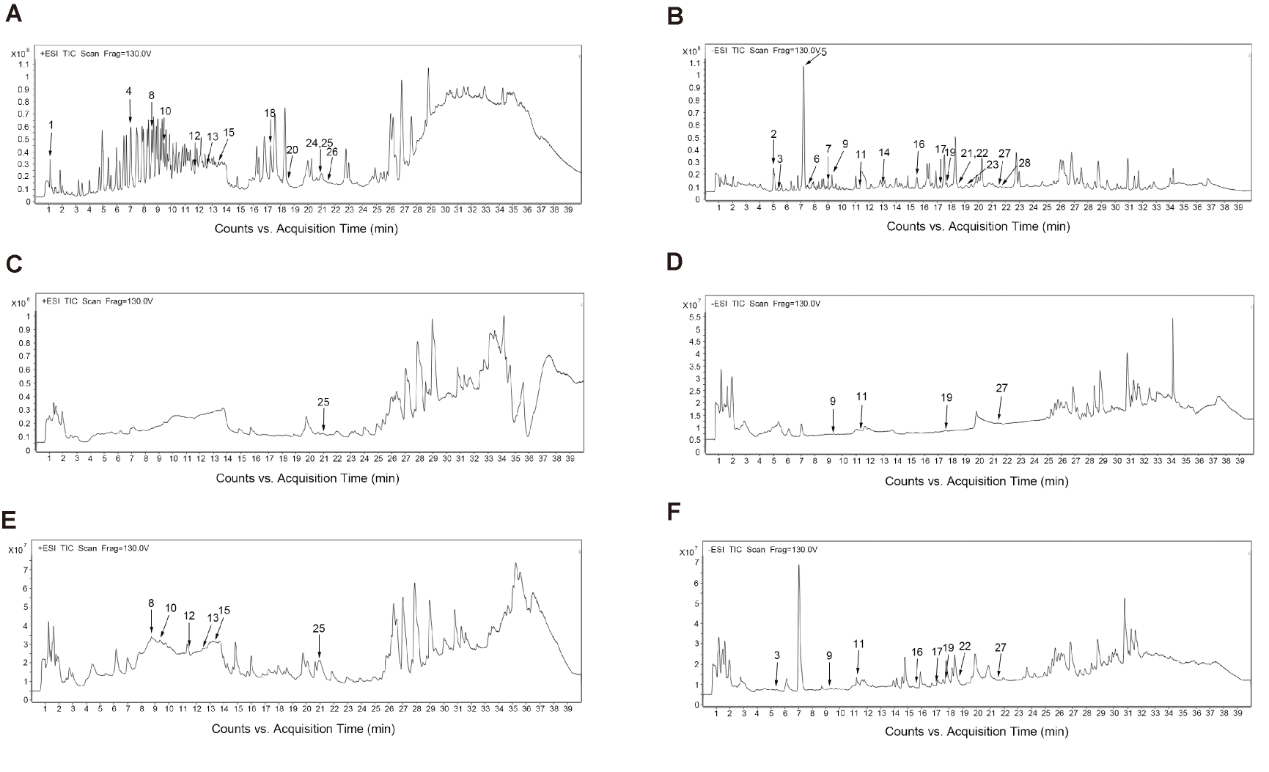
**

**Figure S7.** The total ion chromatography in positive and negative modes for rat serum (**A**, **B**), heart (**C**, **D**), and liver (**E**, **F**) samples by UHPLC-Q-TOF/MS.

Compounds 1-28 were listed in table S3.

**
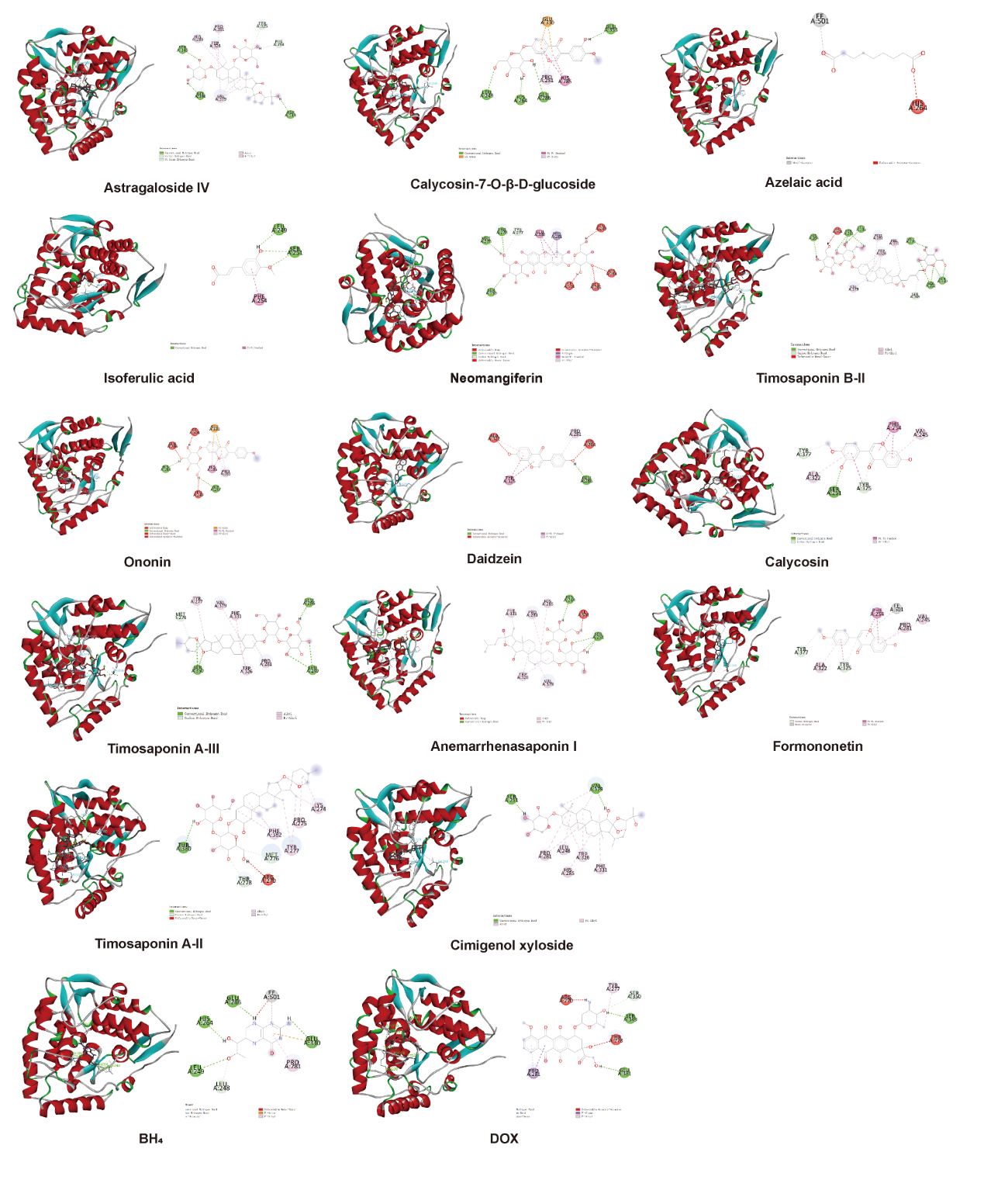
**

**Figure S8.** Molecular docking of PAH with hepatic active compounds.


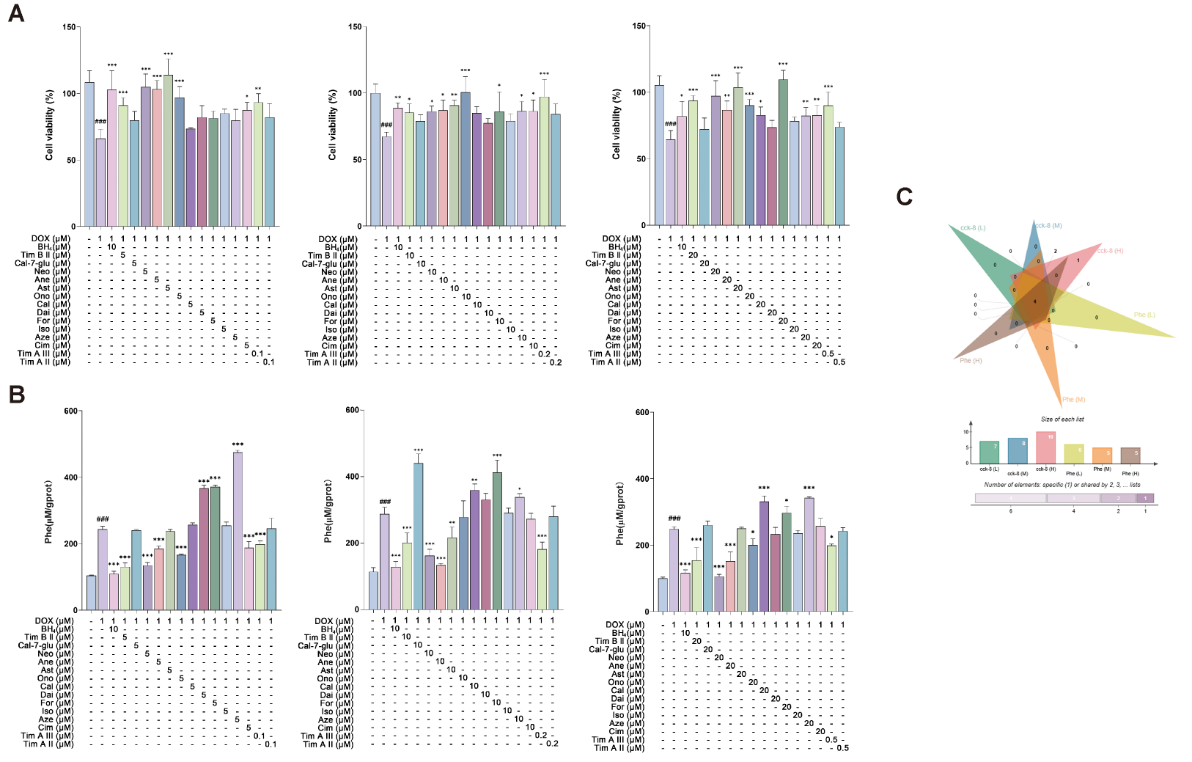


**Figure S9.** Timosaponin B-II, neomangiferinn, anemarrhenasaponin I and timosaponin A-III could inhibit the DOX-induced cell injury

(**A**-**C**) AML12 cells were treated with DOX (1 μM, 24 h) and three concentration gradients for compounds from SXT, cells survival rate detected by CCK8; (**D**-**F**) phenylalanine levels when AML12 cells were treated with DOX (1 μM, 24 h) and three different concentration gradients of compounds from SXT; (**G**) Venn chart deciphered the four compounds could decrease phenylalanine level. Tim B II: timosaponin B II; Cal-7-glu: calycosin-7-O-glu; Neo: neomangiferin; Ane: anemarrhenasaponin I; Ast: astragaloside IV; Ono: ononin; Cal: calycosin; Dai: daidzein; For: formononetin; Iso: isoferulic acid; Aze: azelaic acid; Cim: cimigenol xyloside; Tim AIII: timosaponin A III; Tim AII: timosaponin A II.
^#^*p* < 0.05, vs. the CON group; **p* < 0.05, ***p* < 0.01, ****p* < 0.001 vs. the DOX group.

**
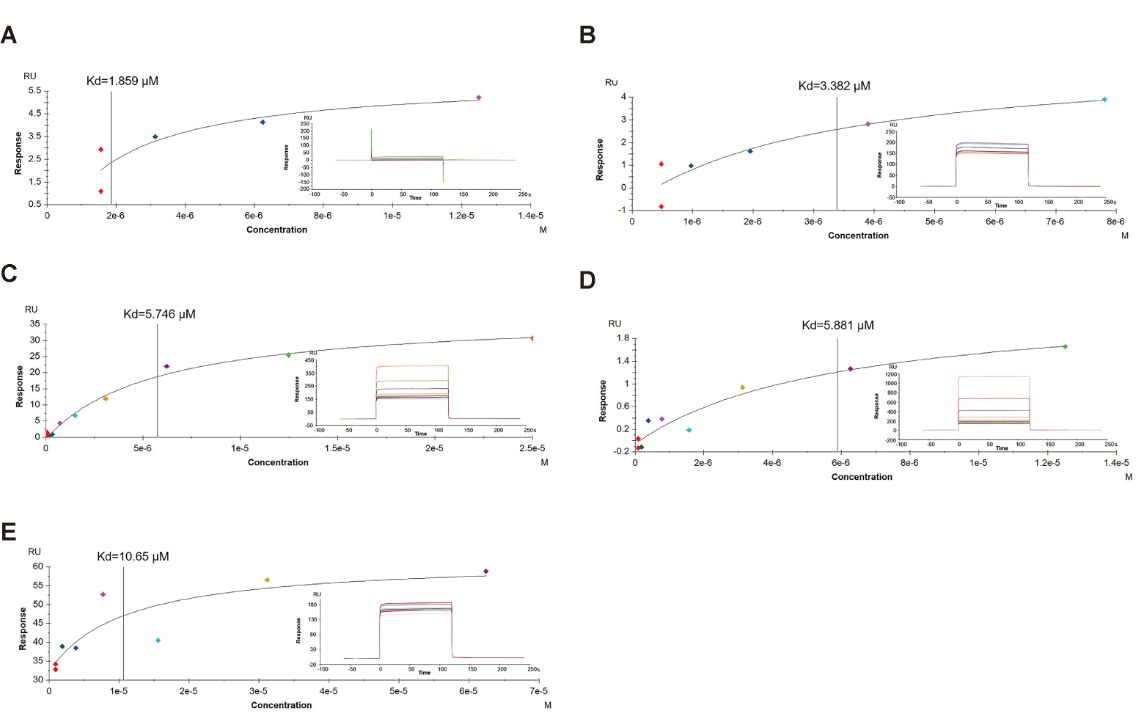
**

**Figure S10.** Binding affinity analysis of PAH with BH_4_ and four compounds from SXT.

(**A**) anemarrhenasaponin I; (**B**) timosaponin B-II; (**C**) timosaponin A-III; (**D**) neomangiferin; (**E**) BH_4_

**
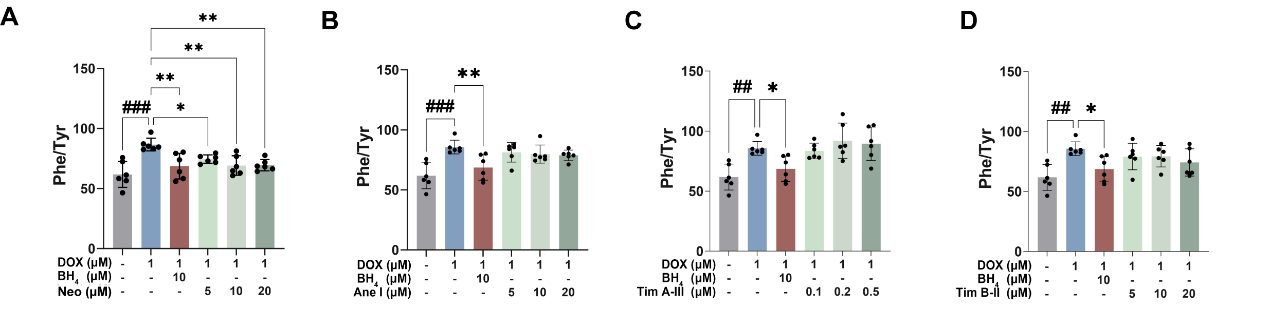
**

**Figure S11.** Anemarrhenasaponin I (**A**), timosaponin A-III (**B**), and timosaponin B-II (**C**) produced no change on the Phe/Tyr ratio in DOX-treated AML12 cells. Phe: phenylalanine, Tyr: tyrosine, Ane I: anemarrhenasaponin I, Tim A-III: timosaponin A-III, Tim B-II: timosaponin B-II.

^##^*p* < 0.01, vs. the CON group; **p* < 0.05, ***p* < 0.01 vs. the DOX group.

**
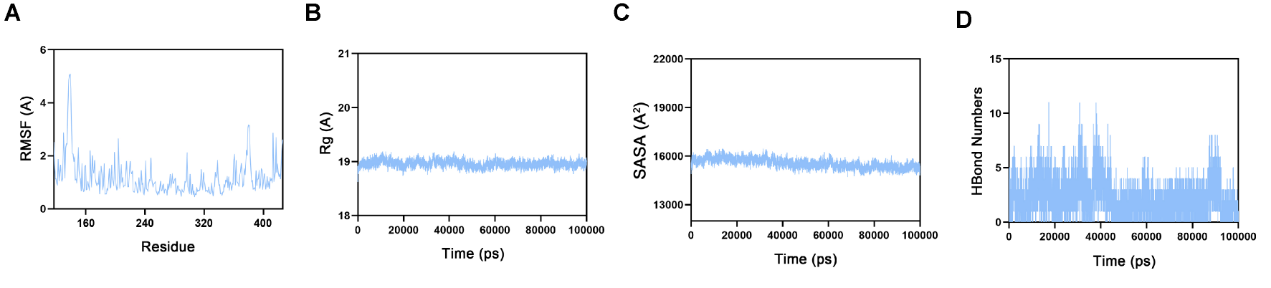
**

**Figure S12.** Visualization analysis of molecular dynamics results. (**A**) RMSF; (**B**) Rg; (**C**) SASA; (**D**) hydrogen bonds analyses.

**3. Supplementary tables**

**Table S1** Composition of SXT

| **Herbs** | **Chinese name** | **Species** | **Medicinal part** | **Origin** | **Batch No.** | **Grams**  **(g)** |
| --- | --- | --- | --- | --- | --- | --- |
| Astragali radix | Huangqi | Astragalus membranaceus (Fisch.) Bunge | Root | Dingxi, Gansu Province | 23090603 | 22.38 |
| Bupleuri Radix | Chaihu | Bupleurum Chinese Dc. | Root | Yuncheng, Shanxi Province | 2301030093 | 5.60 |
| Anemarrhenae Rhizoma | Zhimu | Anemarrhena asphodeloides Bge. | Root and rhizomes | Bozhou, Anhui Province | 24020201 | 11.19 |
| Cimicifugae Rhizoma | Shengma | Cimicifuga heracleifolia Kom. | Root and rhizomes | Anshan, Liaoning Province | 21070611 | 3.73 |
| Platycodonis Radix | Jiegeng | Platycodon grandiflorus(Jacq.)A.Dc. | Root | Bozhou, Anhui Province | 23102502 | 5.60 |

**Table S2** SXT key information form

| SXT key information form issued by National Administration of Traditional Chinese Medicine | |
| --- | --- |
| 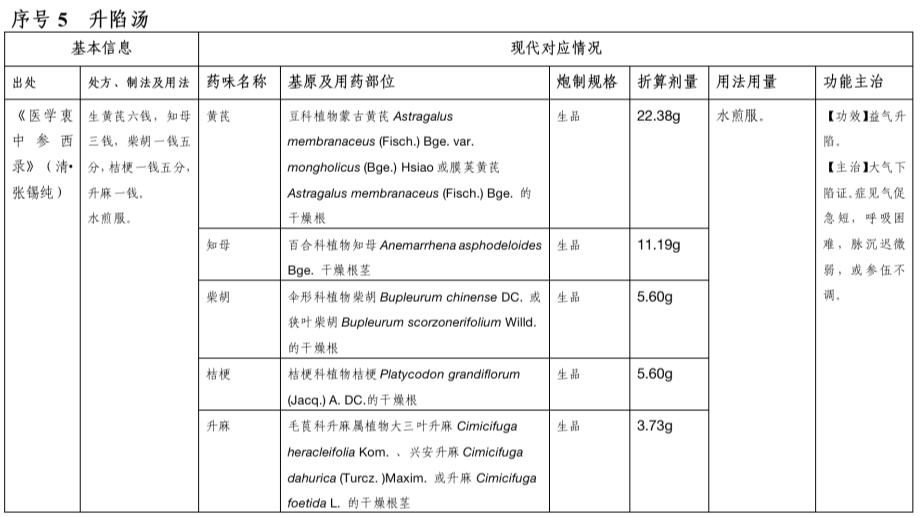 |  |

**Table S3** Metabolic pathways with significant trends (P < 0.05).

| **Pathway Name** | **Match Status** | ***P* value** | **-log(p)** | **Impact** |
| --- | --- | --- | --- | --- |
| Phenylalanine metabolism | 5 | 0.000246 | 3.6090 | 0.61904 |
| Phenylalanine, tyrosine and tryptophan biosynthesis | 3 | 0.000595 | 3.2252 | 1 |
| Tryptophan metabolism | 6 | 0.020838 | 1.6811 | 0.05583 |
| Arginine biosynthesis | 3 | 0.036458 | 1.4382 | 0.11675 |

**Table S4** The blood, heart and hepatic absorbed constituents from SXT in the DOX-induced CHF rats.

|  | **Herbs** | **Serum** | **Heart** | **Liver** |
| --- | --- | --- | --- | --- |
| 1 | Astragali radix | Trigonelline* | - | - |
| 2 | Cimicifugae Rhizoma | piscidic acid | - | - |
| 3 | Anemarrhenae Rhizoma | neomangiferin* | - | neomangiferin* |
| 4 | Cimicifugae Rhizoma | prim-O-glucosylcimifugin* | - | - |
| 5 | Anemarrhenae Rhizoma | mangiferin* | - | - |
| 6 | Anemarrhenae Rhizoma | isomangiferin | - | - |
| 7 | Astragali radix | coumaric acid | - | - |
| 8 | Astragali radix | calycosin-7-O-β-D-glucoside* | - | calycosin-7-O-β-D-glucoside* |
| 9 | Astragali radix | azelaic acid | azelaic acid | azelaic acid |
| 10 | Cimicifugae Rhizoma | isoferulic acid* | - | isoferulic acid* |
| 11 | Anemarrhenae Rhizoma | timosaponin B-II* | timosaponin B-II* | timosaponin B-II* |
| 12 | Astragali radix | Ononin* | - | Ononin* |
| 13 | Astragali radix | daidzein* | - | daidzein* |
| 14 | Platycodonis Radix | platycodin D* | - | - |
| 15 | Astragali radix | calycosin* | - | calycosin* |
| 16 | Astragali radix | astragaloside IV* | - | astragaloside IV* |
| 17 | Anemarrhenae Rhizoma | anemarrhenasaponin I | - | anemarrhenasaponin I |
| 18 | Astragali radix | astrapterocarpan* | - | - |
| 19 | Astragali radix | formononetin* | formononetin* | formononetin* |
| 20 | Anemarrhenae Rhizoma | gitogenin | - | - |
| 21 | Bupleuri Radix | saikosaponin A* | - | - |
| 22 | Anemarrhenae Rhizoma | timosaponin A-II | - | timosaponin A-II |
| 23 | Bupleuri Radix | saikosaponin B2* | - | - |
| 24 | Cimicifugae Rhizoma | 7,8-didehydrocimigenol | - | - |
| 25 | Cimicifugae Rhizoma | cimigenol xyloside* | cimigenol xyloside* | cimigenol xyloside* |
| 26 | Anemarrhenae Rhizoma | parigenin | - | - |
| 27 | Anemarrhenae Rhizoma | timosaponin A-III* | timosaponin A-III* | timosaponin A-III* |
| 28 | Anemarrhenae Rhizoma | timosaponin A-IV | - | - |

* means confirmed by comparison with reference standards.

**Table S5** The affinities of PAH with BH_4_ and neomangiferin

| **Compounds** | **Formula** | **KD (μM)** | **Rmax (RU)** | **offset (RU)** | **Chi2 (RU2)** |
| --- | --- | --- | --- | --- | --- |
| anemarrhenasaponin I | C_39_H_66_O_14_ | 1.859 | 7.43 | -1.368 | 0.919 |
| timosaponin B-II | C_45_H_76_O_19_ | 3.382 | 6.507 | -0.6688 | 0.623 |
| timosaponin AIII | C_39_H_64_O_13_ | 5.746 | 38.66 | -0.5265 | 1.5 |
| neomangiferin | C_25_H_28_O_16_ | 5.881 | 2.537 | -0.05829 | 0.0362 |
| BH_4_ | C_9_H_15_N_5_O_3_ | 10.65 | 29.74 | 32.18 | 33.4 |

KD (μ M), dissociation equilibrium constant; Rmax (RU), maximum response value; Offset (RU), offset value; Chi2 (RU2), chi square value.
